# Reference genomes and transcriptomes of *Nicotiana sylvestris *and *Nicotiana tomentosiformis*

**DOI:** 10.1186/gb-2013-14-6-r60

**Published:** 2013-06-17

**Authors:** Nicolas Sierro, James ND Battey, Sonia Ouadi, Lucien Bovet, Simon Goepfert, Nicolas Bakaher, Manuel C Peitsch, Nikolai V Ivanov

**Affiliations:** 1 Philip Morris International R&D, Philip Morris Products SA, Quai Jeanrenaud 5, 2000 Neuchatel, Switzerland

## Abstract

**Background:**

*Nicotiana sylvestris *and *Nicotiana tomentosiformis *are members of the Solanaceae family that includes tomato, potato, eggplant and pepper. These two *Nicotiana *species originate from South America and exhibit different alkaloid and diterpenoid production. *N. sylvestris *is cultivated largely as an ornamental plant and it has been used as a diploid model system for studies of terpenoid production, plastid engineering, and resistance to biotic and abiotic stress. *N. sylvestris *and *N*. *tomentosiformis *are considered to be modern descendants of the maternal and paternal donors that formed *Nicotiana tabacum *about 200,000 years ago through interspecific hybridization. Here we report the first genome-wide analysis of these two *Nicotiana *species.

**Results:**

Draft genomes of *N. sylvestris *and *N. tomentosiformis *were assembled to 82.9% and 71.6% of their expected size respectively, with N50 sizes of about 80 kb. The repeat content was 72-75%, with a higher proportion of retrotransposons and copia-like long terminal repeats in *N. tomentosiformis*. The transcriptome assemblies showed that 44,000-53,000 transcripts were expressed in the roots, leaves or flowers. The key genes involved in terpenoid metabolism, alkaloid metabolism and heavy metal transport showed differential expression in the leaves, roots and flowers of *N*. *sylvestris *and *N. tomentosiformis*.

**Conclusions:**

The reference genomes of *N. sylvestris *and *N. tomentosiformis *represent a significant contribution to the SOL100 initiative because, as members of the *Nicotiana *genus of Solanaceae, they strengthen the value of the already existing resources by providing additional comparative information, thereby helping to improve our understanding of plant metabolism and evolution.

## Background

Woodland tobacco (*Nicotiana sylvestris*) grows naturally in the Andes from Bolivia to Argentina and is largely cultivated nowadays as an ornamental plant. *Nicotiana tomentosiformis *also grows naturally in the Andes but over a wider range, from Peru to Argentina [1]. *N. sylvestris *(2n = 24) and *N. tomentosiformis *(2n = 24) belong to clades of the *Nicotiana *sections Sylvestres and Tomentosae, respectively, of the Solanaceae family, which have diverged about 15 million years ago [2]. Other members of this family include many agriculturally important species such as tomato, potato, eggplant and pepper. *N. sylvestris *is considered to be the maternal donor, which about 200,000 years ago merged through interspecific hybridization with *N. tomentosiformis *(most likely paternal donor) to form an allotetraploid *N. tabacum *(2n = 4x = 48), the common tobacco [3]. Thus, the *N. sylvestris *and *N. tomentosiformis *genome sequences are expected to have high identity to the S-genome and T-genome of *N. tabacum*, respectively. Both are important for understanding the biological processes - for example, regulation of gene expression, in allotetraploid *N. tabacum *species.

*N. sylvestris *and *N. tomentosiformis *are diploid species with an estimated 1C genome size of about 2,650 Mb. As summarized in the Plant DNA C-values database [4], the genome size estimation based on 1C measurements for *N. sylvestris *ranges from 2.078 to 2.812 Gb [3,5-9], with the generally accepted size of 2.636 Gb. For *N. tomentosiformis*, the genome size ranges from 1.809 to 2.763 Gb [3,7,8], with the accepted size of 2.682 Gb.

A subset of simple sequence repeat (SSR) markers derived from the Tobacco Genome Initiative [10] and conserved ortholog set (COSII) [11] was used to construct a genetic map for the diploid *N. tomentosiformis *(on a mapping population of *N. tomentosiformis *TA3385 × *N. otophora *TA3353) and for *N. acuminata*, a species closely related to *N. sylvestris *[12]. It was because of the failure to produce a suitable mapping population for *N. sylvestris *that a mapping population of *N. acuminata *TA3460 × *N. acuminata *TA3461 was used instead. A high density genetic map of an allotetraploid *N. tabacum *was built based on a complete set of 2,317 SSR markers applied to an F2 mapping population of Hicks Broadleaf and Red Russian [13]. Recently, another genetic map of tobacco was constructed from SSR markers applied to a mapping population of two flue-cured tobacco varieties, Honghua Dajinyuan and Hicks Broadleaf [14]. All these genetic markers can serve as anchoring points for validation of the *N. sylvestris *and *N. tomentosiformis *genome assemblies because of their high similarity to the S- and T-genomes of tobacco.

In plant biology, *N. sylvestris *serves as a diploid model system for studies of terpenoid production in glandular trichomes [15,16], engineering of plastid genomes [17,18], mitochondrial function [19,20], herbicide resistance [21,22] and plant virus resistance [23]. Besides its contribution to tobacco, *N. sylvestris *has been shown to be a modern descendent of one of the progenitors of other polyploid *Nicotiana *species (for example, *N. nudicaulis, N. repanda, N. nesophila *and *N. stocktonii*) [1]. Chase *et al. *[24] have even suggested that *N. sylvestris *might have been 'ancestral' to all the *Nicotiana *species because it easily produces crosses within the genus. *N. tomentosiformis *has been investigated mostly as a possible ancestor of *N. tabacum*.

Similar to other members of the Solanaceae family, *N. sylvestris *produces a wide range of alkaloids that are toxic to insects; this serves as a well-established mechanism of its natural defense against damage to leaves and flowers caused by herbivores [25]. Leaves of the field-grown plants under a defoliation regime exhibit a four-fold increase in total leaf alkaloids depending on leaf position compared with undamaged control plants [26]. It was shown that wounding induces nicotine transport to the injured tissues via the jasmonate signal transduction pathway [25]. Upon wounding, accumulated jasmonates in the shoots are transported to the roots, where they stimulate nicotine synthesis leading to augmented nicotine availability throughout the plant [27]. Nicotine is the predominant alkaloid in *N. sylvestris *[28] and, unlike for most *Nicotiana *species in which the roots contain higher quantities of alkaloids compared with the leaves, the total alkaloid content in dry *N. sylvestris *leaves is the highest (2.96%) in the genus and only 0.786% in roots [29]. The nicotine content of *N. sylvestris *(82% of 4.8 mg/g total alkaloids) was found to be much higher than the nicotine content of *N. tomentosiformis *(6% of 0.5 mg/g total alkaloids), and this could be the driving force behind the favorable allotetraploidization between *N. sylvestris *and other *Nicotiana *species [30]. Nornicotine is the predominant alkaloid (65% of 0.5 mg/g total alkaloids) in *N. tomentosiformis*, which is typical for the members of Tomentosae section. In this work, we provide a genomic explanation for the dramatic differences in the alkaloid metabolism between these two *Nicotiana *species.

The *Nicotiana *genus is a rich source of terpenoids, the biosynthesis of which has been reviewed previously [31,32]. Terpenoids play a significant role as attractants to a number of insects that pollinate *N. sylvestris *and *N. tomentosiformis *[27]. Two of the most abundant diterpenoids, cembranoids and labdanoids, are synthesized in the trichome glands of *N. tabacum *cultivars. However, *N. sylvestris *produces predominantly labdanoids and *N. tomentosiformis *produces predominantly cembranoids [27,33]. To better understand these differences at the genomic level, we attempted to investigate the structure and copy number of the genes responsible for diterpenoid metabolism in their respective genomes.

Another peculiar property of *Nicotiana *species is their high susceptibility to accumulate cadmium as well as other heavy metals [34]. Rosén *et al. *[35] compared the availability of added and naturally occurring soil cadmium in *N. sylvestris *plants and found that cadmium concentrations in the leaves was three-fold higher than in the roots, and two-fold higher than in the soil. We examined a set of genes believed to be involved in heavy metal accumulation and describe their structural variations between the two *Nicotiana *plants.

High quality genome sequences of tomato and potato have been published and annotated thoroughly by the Solanaceae community [36,37]. Comparison of the *N. sylvestris *and *N. tomentosiformis *genomes with these two reference genomes will improve our understanding of *Nicotiana *clade evolution and expedite the functional annotation of their genomes. A draft of the first *Nicotiana *genome (*N. benthamiana*, about 3 Gb in size) has recently been published and its utility has been shown immediately in the discovery of homologs of some immunity-associated genes [38]. Because the allotetraploid genome of *N. benthamiana *is a result of the hybridization of two *Nicotiana *species at least one of which is thought to be a member of the Sylvestres clade [30,39], a certain degree of synteny with the genome of *N. sylvestris *might be expected.

The estimated genome sizes of *N. sylvestris *and *N. tomentosiformis *(approximately 2,600 Mb) are nearly three times larger than the tomato (900 Mb) [37] or potato (844 Mb) [36] genomes, a phenomenon that could be explained by repeat expansion in the *Nicotiana *genomes due to the accumulation of transposable elements. C_0_t measurements in the *N. tabacum *genome, which showed the presence of 55% short (approximately 300 nucleotides) and 25% long (approximately 1,500 nucleotides) repeats [40], support this hypothesis. Similarly, pepper euchromatin doubled its size compared with tomato through a massive gain of a specific group of long terminal repeat (LTR) retrotransposons [41]. The *N. sylvestris *genome showed signs of more recent repeat expansions with higher homogeneity, whereas the genome of *N. tomentosiformis *showed significantly higher repeat diversity [2]. Further, the *N. sylvestris *genome was reported to have a higher content of Tnt1 transposons and a more uniform distribution of the elements than the *N. tomentosiformis *genome [42,43]. A more detailed analysis showed that the relative copy numbers of four retrotransposons (Tnt1-OL13, Tnt1-OL16, Tnt2d and Tto1-1R) were higher in *N. sylvestris *than in *N. tomentosiformis *[44]. Conversely, Renny-Byfield *et al. *[45] found that a highly repetitive DNA sequence (NicCL3) made up to 2% of the *N. tomentosiformis *genome but was almost absent in the *N. sylvestris *genome. A second repetitive DNA sequence, NicCL7/30, was also found to be more represented in *N. tomentosiformis *than *N. sylvestris*, although not as strongly. Other repeat families, EPRV [46], TAS49 [47] and GRS [43], were identified in both genomes and displayed differential copy number, distribution and methylation patterns. These findings emphasize the significant differences in the evolution of the two genomes since they diverged about 15 million years ago.

The assembly of the *N. sylvestris *and *N. tomentosiformis *transcriptomes based on 454 sequencing data showed that only 33% of the sequences contained substitutions between the two species [48]. Bombarely *et al. *[48] suggested that additional Illumina sequencing of the transcriptome should overcome the 'homopolymer' problem due to pyrosequencing and that genomic DNA sequencing would allow an increased number of SNPs to be identified. Elucidating the transcriptomes of *N. sylvestris *and *N. tomentosiformis *can shed light on their protein complement, and allow more targeted experimental investigations of these and related species. Recently an Affymetrix Tobacco Exon Array was developed based on the current genome and EST sequence data from the Tobacco Genome Initiative, which cover a large proportion of the tobacco gene space [49]. Because the probes that cover both the S-genome and T-genome of *N. tabacum *are very similar to the *N. sylvestris *and *N. tomentosiformis *genomes, respectively; in this study we have used the Tobacco Exon Array to investigate the differential gene expression between the latter two *Nicotiana *species.

Here, we present the sequencing and assembly of the *N. sylvestris *and *N. tomentosiformis *whole genomes as well as the transcriptomes from leaves, roots and flowers. We assess the assembly quality, and analyze and compare them to the existing genomes and transcriptomes from other members of the Solanaceae family. We take a more detailed look at the gene families involved in alkaloid and terpenoid metabolism and heavy metal transport because they should contribute to the unique characteristics of these two plants.

## Results and discussion

### Genome assembly

The *N. sylvestris *and *N. tomentosiformis *genomes were sequenced using a whole-genome shotgun sequencing approach. For *N. sylvestris*, a 94× coverage of 100 bp Illumina HiSeq-2000 reads was used. In total, six libraries were constructed with different insert sizes ranging from 180 bp to 1 kb for paired-end libraries, and from 3 to 4 kb for mate-pair libraries. The numbers of clean reads in each library are summarized in Additional file 1. Similarly, for *N. tomentosiformis *a 146× coverage of 100 bp Illumina HiSeq-2000 reads was used. In total, seven libraries were constructed with different insert sizes ranging from 140 bp to 1 kb for paired-end libraries, and from 3 to 5 kb for mate-pair libraries. The numbers of clean reads in each library are summarized in Additional file 2.

The genomes were assembled by creating contigs from the paired-end reads and then scaffolding them with the mate-pair libraries. In this step, mate-pair information from closely related species was also used. The resulting final assemblies, described in table 1, amounted to 2.2 Gb and 1.7 Gb for *N. sylvestris *and *N. tomentosiformis*, respectively, of which, 92.2% and 97.3% were non-gapped sequences. The *N. sylvestris *and *N. tomentosiformis *assemblies contain 174 Mb (7.8%) and 46 Mb (2.7%) undefined bases, respectively. The *N. sylvestris *assembly contains 253,984 sequences, its N50 length is 79.7 kb, and the longest sequence is 698 kb. The *N. tomentosiformis *assembly is made of 159,649 sequences, its N50 length is 82.6 kb, and the longest sequence is 789.5 kb.

**Table 1:** Statistics of the assembly of the *N.*

	*N. sylvestris*	*N. tomentosiformis*
Sequences	253,984	159,649
Average length (bp)	8,748.83	10,576.84
Maximum length (bp)	698,072	789,565
N50 length (bp)	79,724	82,598
Total length (bp)	2,222,062,302	1,688,581,715
Undefined bases	174,351,674 (7.8%)	45,955,292 (2.7%)
Genome coverage	82.9%	71.6%

*sylvestris *and *N. tomentosiformis *draft genomes

With the advent of next-generation sequencing, genome size estimations based on k-mer depth distribution of sequenced reads are becoming possible [36,50-52]. For instance, the recently published potato genome was estimated to be 844 Mb using a 17-mer distribution [36], in good agreement with its 1C size of 856 Mb [4]. Furthermore, the analysis of repetitive content in the 727 Mb potato genome assembly and in bacterial artificial chromosomes and fosmid end sequences indicated that much of the unassembled genome sequences were composed of repeats [36]. In *N. sylvestris *and *N. tomentosiformis *the genome sizes were estimated by this method using a 31-mer to be 2.68 Gb and 2.36 Gb, respectively. While the *N. sylvestris *estimate is in good agreement with the commonly accepted size of its genome based on 1C DNA values, the *N. tomentosiformis *estimate is about 15% smaller than its commonly accepted size. Estimates using a 17-mer were smaller; 2.59 Gb and 2.22 Gb for *N. sylvestris *and *N. tomentosiformis*, respectively. Using the 31-mer depth distribution, we estimated that our assembly represented 82.9% of the 2.68 Gb *N. sylvestris *genome and 71.6% of the 2.36 Gb *N. tomentosiformis *genome.

The proportion of contigs that could not be integrated into scaffolds was low; namely, the *N. sylvestris *assembly contains 59,563 contigs (18 Mb; N50 length of 273 bp) that were not integrated in scaffolds, and the *N. tomentosiformis *assembly contains 47,741 contigs (17.3 Mb; N50 length of 346 bp) that were not integrated in scaffolds. Using the regions of the Whole Genome Profiling (WGP) physical map of tobacco [53] that are of *N. sylvestris *or *N. tomentosiformis *ancestral origin, the assembly scaffolds were superscaffolded and an N50 of 194 kb for *N. sylvestris *(10,261 contigs and scaffolds in 2,637 superscaffolds) and of 166 kb for *N. tomentosiformis *(7,463 contigs and scaffolds in 1,989 superscaffolds) were obtained. Superscaffolding was performed using the WGP physical map contigs as templates and positioning the assembled sequences for which an orientation in the superscaffolds could be determined. This approach discards any anchored sequence of unknown orientation as well as any sequence that spans across several WGP contigs, thereby reducing the number of superscaffolded sequences. Furthermore, the superscaffolding introduced additional unknown bases (N) into the assembly because the length of each stretch was estimated based on the tobacco genome.

### Repeat content

The repeat content of the *N. sylvestris *and *N. tomentosiformis *genomes is summarized in Table 2. Additional file 3 shows this in more detail. More than 70% of both genomes are repeat elements. In *N. tomentosiformis*, there seem to be more copia-type LTRs (13.43% and 9.13%, respectively) and retrotransposons (13.05% and 10.33%, respectively) than in *N. sylvestris*; while the amount of gypsy-like LTRs is about 20% in both genomes. The difference between the total size of sequenced DNA and repeat-masked DNA indicates that the gene-rich DNA is around 625 Mb for *N. sylvestris *and 425 Mb for *N. tomentosiformis*.

**Table 2:** Composition of the repeat elements in the *N*.

	*N. sylvestris*		*N. tomentosiformis*	
		
Repeat element	Number of bases	%	Number of bases	%
LINE	5,828,979	0.27	2,834,174	0.17
SINE	4,040,138	0.18	5,244,169	0.31
LTR/copia	203,592,581	9.13	227,491,087	13.43
LTR/gypsy	463,070,166	20.75	343,784,620	20.32
LTR/others	184,881,207	8.28	90,166,206	5.33
Transposons	33,621,895	1.51	22,593,004	1.34
Retrotransposons	230,653,066	10.33	220,727,245	13.05
Simple repeats	4,954,900	0.22	4,809,855	0.28
Low complexity	10,145,060	0.45	9,723,109	0.57
Others	95,243,945	4.28	130,185,895	7.72
Unknown	197,792,439	8.86	116,127,639	6.86
**Total**	**1,605,541,978**	**71.95**	**1,266,206,541**	**74.84**

*sylvestris *and *N. tomentosiformis *genomes

More Tnt1 retrotransposons are found in *N. tomentosiformis *than in *N. sylvestris*, (7.39% and 3.98% respectively), which apparently contradicts previous reports [42-44]. This finding could be caused by the mislabeling of novel *N. tomentosiformis *repetitive elements obtained by RepeatScout as Tnt1. The amounts of Tnt2 and Tto1 repetitive elements are higher in *N. sylvestris *than in *N. tomentosiformis *and this finding agrees with previous studies. Furthermore, as reported previously [45], we also observed a higher proportion of NicCL3 (0.75% versus 0.14%) and NicCL7/30 (5.57% versus 2.94%) repetitive DNA elements in *N. tomentosiformis *than in *N. sylvestris*.

### Genetic markers

The 2,363 tobacco SSR markers reported previously [13] were mapped to both genome assemblies. The number of uniquely mapped markers on each genome was then compared with the results of the PCR amplification tests performed in *N. sylvestris *and *N. tomentosiformis*, in order to assign an origin to them when creating the tobacco genetic map (Additional file 4). Sixty-five percent of the SSR markers that amplified only in *N. sylvestris *mapped only to the *N. sylvestris *genome; 7% mapped to both genomes. Similarly, 65% of the SSR markers that amplified only in *N. tomentosiformis *mapped only to *N. tomentosiformis*; 15% mapped to both *N. sylvestris *and *N. tomentosiformis*. About a third of the tobacco SSR markers could not be mapped. This can be expected, because the current draft genome assemblies are likely to fail assembling in regions with simple repeats such as the ones found in SSR markers. If this is the case, a primer pair will match to two different sequences.

Of the 173 SSR markers present in the *N. acuminata *genetic map (Additional file 5), 128 (74%) of them could be mapped to the *N. sylvestris *genome assembly. This number is the sum of the 75 SSRs of the *N. acuminata *map found in the *N. sylvestris *assembly, the 50 SSRs of the *N. acuminata *map found in the *N. sylvestris *and *N. tomentosiformis *assemblies, the single SSR of the *N. acuminata *and *N. tomentosiformis *maps found in the *N. sylvestris *assembly, and the 2 SSRs of the *N. acuminata *and *N. tomentosiformis *maps found in the *N. sylvestris *and *N. tomentosiformis *assemblies (Additional file 6). Similarly, of the 221 SSR markers present in the *N. tomentosiformis *genetic map (Additional file 7), 173 (78%) could be mapped to the *N. tomentosiformis *genome assembly (Additional file 6). In addition, 706 SSR markers not present on the existing genetic maps could be mapped to the *N. sylvestris *genome assembly, 605 mapped to the *N. tomentosiformis *genome assembly, and 174 mapped to both.

Of the 134 COSII markers present in the *N. acuminata *genetic map, 45 (34%) could be mapped to the *N. sylvestris *genome assembly (Additional file 8). Similarly, of the 262 COSII markers in the *N. tomentosiformis *genetic map, 81 (31%) could be mapped to the *N. tomentosiformis *genome assembly (Additional file 8). Using the same method, 736 of the 879 COSII markers (84%) on the expen2000 tomato genetic map could be found; 718 of them mapped to the expected chromosome. In addition, 68 COSII markers not present on the existing genetic maps could be mapped to the *N. sylvestris *genome assembly, 78 mapped to the *N. tomentosiformis *genome assembly, and 226 mapped to both.

The low numbers of COSII markers that could be mapped to the *N. sylvestris *and *N. tomentosiformis *assemblies, despite the good results that were obtained using the same method on the tomato map, could be due to the current fragmented state of the assemblies, or because the COSII marker primers are not adapted for *Nicotiana *species.

### Transcriptome assembly

The number of reads obtained for each of the tissue-specific samples from both species is outlined in Additional file 9. Tissue-specific assemblies were generated for the three samples (root, leaf and flower) by mapping the reads to the reference genomes using the Bowtie2/Tophat2 pipeline. The length distributions of the assembled transcripts are summarized in table 3. In addition, a reference transcriptome for each species was created by merging the three individual tissue-specific assemblies. We also used a *de novo *assembly program to generate an assembly that potentially contains transcripts missing from the mapping assembly because of the absence of certain genes from the current reference genome assembly. The size and length distribution of the assembled transcripts is shown in Additional file 10.

**Table 3:** Number and length distribution of transcripts from the tissue-specific read mapping using Cufflinks2

		Sequence	Transcript length distribution				
			
Species	Tissue	number	Q0	Q1	Q2	Q3	Q4
*N. sylvestris*	Flower	53,247	63	796	1,327	2,122	24,850
	Leaf	46,114	72	801	1,372	2,216	23,553
	Root	46,313	72	826	1,364	2,159	20,215
							
*N. tomentosiformis*	Flower	48,043	75	864	1,410	2,200.5	15,607
	Leaf	43,743	89	817	1,415	2,249	19,133
	Root	44,169	69	832	1,388	2,169	16,753

Sequences that contained the pseudo-base N were excluded. Quartiles are labeled with Q0 to Q4.

### Transcript and protein quality

**Figure 1:**
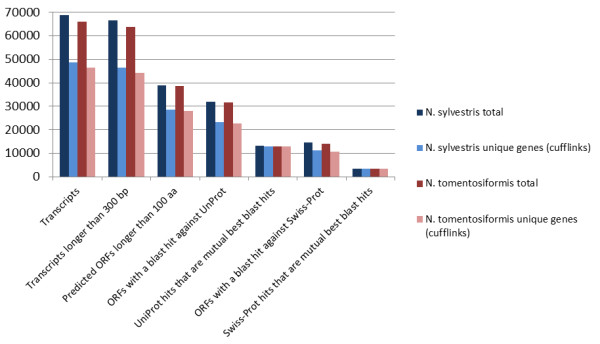
**Numbers of transcripts from the merged tissue assemblies with hits to UniProt plant sequences**.

**Figure 2:**
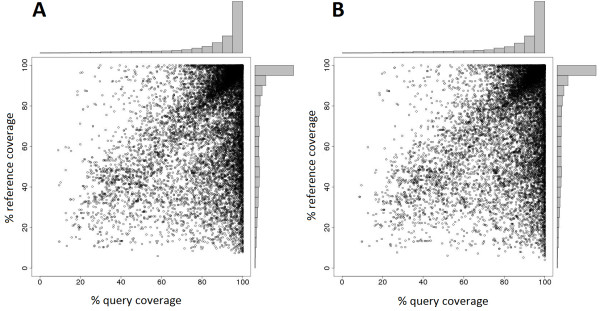
**Percentage coverage of predicted ORFs from the merged transcript assemblies by homologs from UniProt**. Hits were determined by BLAST searches. **(a,b) **The ORFs were derived from the transcripts from the *N. sylvestris *(a) and *N. tomentosiformis *(b) genome assemblies. Dots are indicating the percentage of coverage of the query and the reference for each BLAST hit. Histograms for the coverage of the query and reference show the categorized number of BLAST hits.

The assembled reference transcriptome was assessed for completeness and accuracy by mapping the transcripts to the UniProt reference plant sequence databases. The number of sequences for both the transcripts and the unique genes from which the transcripts are derived that could be mapped was similar for *N. sylvestris *and *N. tomentosiformis *(Figure 1). For *N. sylvestris *and *N. tomentosiformis*, 58.6% and 60.5% of transcripts, respectively, had significant ORFs with a length equal to or longer than 100 amino acids. The majority, 82.2% for *N. sylvestris *and 81.9% for *N. tomentosiformis*, had a homologous sequence in the UniProt Knowledgebase. Approximately a third of these peptide sequences, 37.2% in *N. sylvestris *and 36.5% in *N. tomentosiformis*, had hits in Swiss-Prot, the annotated subset of UniProt. The BLAST alignments show that while the coverage of the predicted ORFs by the reference sequences is generally high (Figure 2) and comparable between the species, the coverage of the reference sequence by the predicted ORFs is often partial, indicating that these ORFs are likely to be incomplete.

### Functional comparison to other species

**Figure 3:**
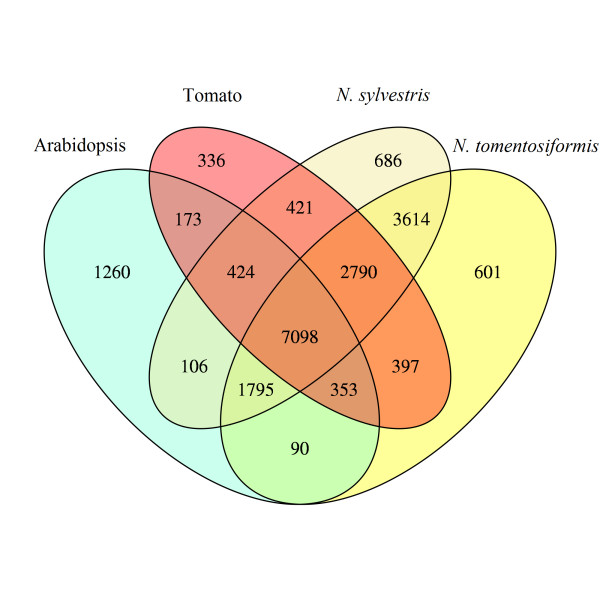
**Clusters of orthologous genes from *N***. *sylvestris*, *N. tomentosiformis*, tomato and *Arabidopsis*. The genes for the predicted *N. sylvestris *and *N. tomentosiformis *ORFs are from the merged transcript assemblies. Tomato is a representative of Solanaceae and *Arabidopsis *is a representative eudicot.

We used the OrthoMCL software [54,55] to define clusters of orthologous and paralogous genes between *N. sylvestris *and *N. tomentosiformis*, as well as tomato, another representative of the Solanaceae family, and *Arabidopsis *as a representative of the eudicots (Figure 3). While a large number of sequences are shared between all the species (7,098), many are specific to Solanaceae (2,790). A very high number of sequences are only observed in the *Nicotiana *species (3,614), with several hundred gene clusters being specific to *N. sylvestris *and *N. tomentosiformis*. These sequences may be artifacts that are the result of incomplete transcripts not clustering correctly, rather than actual novel protein families that evolved since the split of the species.

**Figure 4:**
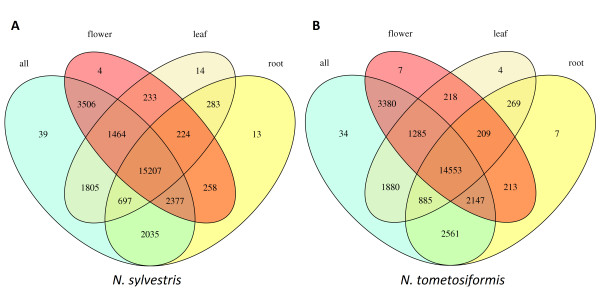
**Clusters of orthologous genes from the individual tissue and merged sample reads**. The gene sequences are derived from RNA-seq reads. **(a) **The gene clusters for *N. sylvestris*. **(b) **The gene clusters for *N. tomentosiformis*.

At the tissue level (Figure 4), the vast majority of gene clusters are shared. As far as the number of clusters is concerned, flowers had the most diverse transcriptome; flowers also contain a large number of transcripts (3,510 for *N. sylvestris *and 3,387 for *N. tomentosiformis*) not found in root or leaf tissues. The number of tissue-specific clusters is very low (<20 for any of the tissues in either of the species); this number reflects the 'noise level' of the merging process because in choosing representative transcripts while merging of the tissue transcriptomes, a different set of exons may have been chosen, and the tissue sequences may not match the representative in the merged transcriptome.

### Functional annotation

Function assignment for proteins was performed by computational means, using the EFICAz program to assign Enzyme Commission (EC) numbers and the InterProScan software to assign Gene Ontology (GO) terms (Table 4).

**Table 4:** Functional annotation of the predicted proteome from the *N. *

	*N. sylvestris *merged	*N. tomentosiformis *merged
Unique IDs	38,940	38,648
		
EC numbers		
Any EC assignment	12,220	11,951
3×EC assignments	7,793	7,611
... to unique proteins	7,554	7,397
3×EC assignments HC	4,117	4,069
...to unique proteins	4,061	4,008
Unique 3×EC numbers	148	150
4×EC assignments	4,427	4,340
...to unique proteins	4,203	4,120
4×EC assignments HC	3,096	3,120
...to unique proteins	2,989	3,007
Unique 4×EC	635	635
		
GO terms		
Unique protein IDs	22,497	22,196
Unique protein IDs with a molecular function	19,852	19,551
Biological process	48,896	48,365
Molecular function	24,680	24,136
Cellular component	8,414	8,419

sylvestris and *N. tomentosiformis *assemblies

EFICAz^2.5 ^was used to assign Enzyme Commission (EC) numbers and InterProScan was used to assign GO terms assignments to the ORFs derived from the assemblies. HC, high confidence.

Over 7,000 proteins could be annotated with a three-digit EC number using the EFICAz tool (7,554 in *N. sylvestris*, 7,397 in *N. tomentosiformis*), of which over 4,000 were assigned with high confidence (4,061 in *N. sylvestris *and 4,008 in *N. tomentosiformis*). This implies that just less than 20% of the predicted proteome of the two species (19.4% and 19.1% for *N. sylvestris *and *N. tomentosiformis*) has enzymatic function. Just over 4,000 and over 3,000 four-digit EC numbers could be assigned to predicted proteins. Although the number of unique four-digit EC numbers is comparatively small (635 for both *N. sylvestris *and *N. tomentosiformis*), this information can still be used to generate molecular pathway databases.

Approximately half of all the proteins were annotated with at least one GO term by the InterProScan software (57.8% for *N. sylvestris *and 57.4% for *N. tomentosiformis*); close to 50,000 biological process tags were assigned and slightly more than 20,000 molecular functions were assigned to just under 20,000 unique proteins. GO term enrichment was analyzed using the GOStats package [56]. Enrichment was tested by comparing the GO term complement for each species against the background of the pooled set of GO terms from both organisms. We see only small and not highly significant changes in gene composition. For *N. sylvestris*, the defense response function is overrepresented; in *N. tomentosiformis *we observe an enrichment of core metabolic functions as well as protein phosphorylation. The phenotypic differences between the species are thus likely to be regulatory rather than due to the loss or gains of new genes. A caveat exists in that the absence of a gene in the genome of one species does not guarantee that the gene does not exist: it is possible that the section of the genome containing the gene simply has not been covered by our current efforts and that further sequencing will identify these. The results of GO term enrichment analysis are shown in Additional file 11.

### Heavy-metal transport

**Figure 5:**
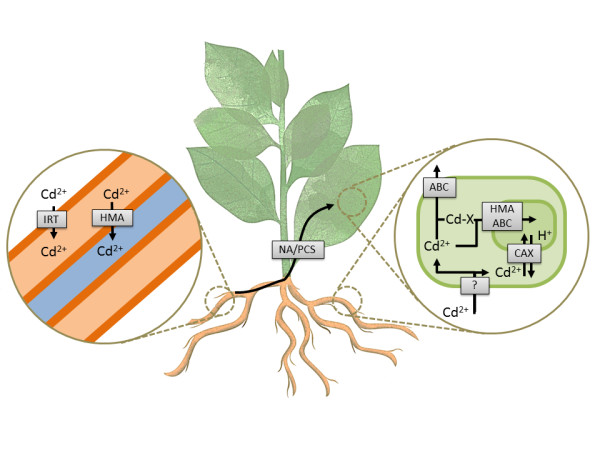
**Diagrammatic representation of heavy-metal transport and accumulation in *Nicotiana *leaves**. Left circle: cadmium is transported by IRT from the environment in the root, and then by HMA into the phloem. Middle: NA and PCS transport cadmium from the root to the leaves. Right circle: in roots and leaves, cadmium enters cells, where it is transported to the vacuole by HMA, ABC or CAX. ABC also exports cadmium outside of the cell. ABC, ATP-binding cassette transporter; CAX, cation/proton exchanger; HMA, heavy metal ATPase; IRT, iron transporter protein; NA, nicotinamine synthase; PCS, phytochelatin synthase.

A non-exhaustive list of gene copies that may be involved in cadmium/zinc (Cd/Zn) accumulation (Figure 5) in *Nicotiana *leaves is shown in Additional file 12. The corresponding transcripts in root, leaf and flower are depicted. The expression data resulting from the hybridization of specific Affymetrix probes (100% matches with the targeted sequences) with leaf RNA isolated from *N. sylvestris *and *N. tomentosiformis *provided data similar to fragments per kilobase of transcript per million mapped reads (FPKM) expression data. The results show that the design of the Affymetrix exon probes is suitable for the analyses of gene expression in both tobacco ancestors, *N. sylvestris *and *N. tomentosiformis*.

Based on sequence and expression data analogies with corresponding *Arabidopsis thaliana *gene data, two *Nicotiana *iron transport-related sequences belonging to the IRT family were identified and named NsylIRT1, NtomIRT1 and NsylIRT2, NtomIRT2 corresponding to *Arabidopsis *IRT1 and IRT2. Both of the *A. thaliana *genes are expressed in the roots and are involved in Zn/Cd uptake [57], although IRT1 is more selective for iron [58]. Interestingly*, IRT1 *and *IRT2 *are expressed in *N. sylvestris *roots but not in *N. tomentosiformis *roots, suggesting that one or more other genes, possibly belonging to the ZIP family, function for Zn and iron uptake in *N. tomentosiformis *[59]. Conversely, the potential *Nicotiana *orthologs (*NtomIRT3 *and *NsylIRT3*) of *AtIRT3 *are not expressed in the roots (Additional file 12), although *AtIRT3 *is expressed in *Arabidopsis *roots, where it is involved in Zn and iron transport [60]. Interestingly, *NsylIRT3 *and *NtomIRT3 *transcripts are more abundant in flower tissues most likely for the redistribution of Zn and Fe. The function of *Nicotiana IRT3 *is possibly closer to the Zrt/IRT-like protein *AtZIP4*, which is highly expressed in anther and pollen [61], where it is suspected to play a role in Zn redistribution in flowers ([62] and references therein). Thus, Zn and iron uptake is likely driven by AtIRT1 and AtIRT2 orthologous proteins in *N. sylvestris*, whereas another gene(s) is likely to perform this function in *N. tomentosiformis*.

The P_1B_-type ATPases, known as heavy metal ATPases (HMAs), play important roles in metal transport in plants. In *Arabidopsis*, AtHMA3 (OsHMA3 in rice) is localized in the tonoplast membrane, where it plays a major role in detoxifying Zn and Cd through vacuolar sequestration. AtHMA3 is recognized as the major locus responsible for the variation in leaf Cd accumulation of *A. thaliana *accessions. AtHMA2 (OsHMA2 in rice) and AtHMA4 (likely OsHMA9 in rice), are localized in the plasma membrane and are expressed in the tissues that surround the vascular vessels of roots, where they function in Zn and Cd efflux from cells [63]. In *N. sylvestris*, *N. tomentosiformis *and *Solanum lycopersicum *(Solyc07g009130) genomes, only one *HMA *gene orthologous to the sub-cluster formed by *AtHMA2*, *AtHMA3 *and *AtHMA4 *in *A. thaliana *is present. This suggests a strong evolutionary divergence between Brassicaceae-Poaceae and Solanaceae. The FPKM expression data show major expression of *Nicotiana *HMA in the root tissues, suggesting that it has functions that are similar to those of AtHMA2, AtHMA3 and AtHMA4, and is more involved in Zn/Co/Cd/Pb translocation from root to shoot than in vacuolar sequestration.

The long-distance root-to-shoot transport of Cd/Zn can be driven by phytochelatins or nicotianamine. Therefore, the key genes that may affect Cd/Zn accumulation in leaves are phytochelatin synthases (PCS) and nicotianamine synthetases (NS) [64,65]. The orthologous genes (*PCS*, *NS1 *and *NS2*) identified in *N. sylvestris *and *N. tomentosiformis *exhibit similar expression profiles in the root, leaf and flower tissues, suggesting that transport in vascular tissues is similar in both *Nicotiana *species.

Genes orthologous to the ABC transporters that are involved in Cd transport in *A. thaliana*, such as *AtPDR8 *(PDR) [66] and *AtATM3 *(ATM) [67], are found in both the *N. sylvestris *and *N. tomentosiformis *genome. Their expression profiles are similar in both *Nicotiana *species and close to their expression profiles in *Arabidopsis*, suggesting that these genes have similar functions in root, leaf and flower in both species. ABC proteins related to the multidrug resistance-associated protein (MRP) family have been already described to be involved in Cd transport and sequestration [68,69] Although the precise cellular function of one of the MRP family members in *N. tabacum*, NtMRP4, has not yet been determined, silencing *NtMRP4 *[70] resulted in Cd reduction in leaves under field conditions. The corresponding gene is expressed in both *N. sylvestris *and *N. tomentosiformis*, suggesting that it has similar functions in both plants.

Other genes that play a role in Cd accumulation into vacuoles belong to the cation proton exchanger (CAX) family. Overexpression of *AtCAX2 *and *AtCAX4 *in tobacco resulted in Cd accumulation in the roots and a subsequent decrease in the shoots [71]. Four genes that clustered with *AtCAX2 *and not *AtCAX4 *(Additional file 13) were identified in *N. sylvestris *and *N. tomentosiformis*, suggesting that tobacco CAX gene products orthologous to AtCAX2 and not AtCAX4 may play roles in Cd sequestration in *Nicotiana *species. The expression profiles of the four genes are similar in both *N. sylvestris *and *N. tomentosiformis*, indicating that these genes play identical functions in both plants.

### Alkaloid metabolism

**Figure 6:**
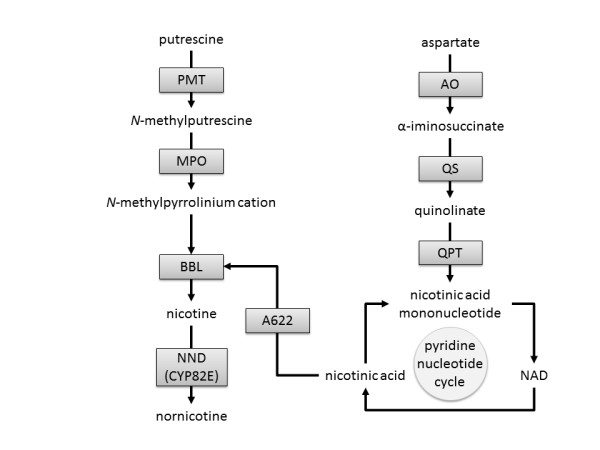
**Key genes involved in the synthesis of nicotine and nornicotine alkaloids in *Nicotiana *leaves**. The berberine bridge enzyme-like protein converts nicotinic acid and *N*-methylpyrrolinium cation into nicotine, and nicotine *N*-deaminase converts it further to nornicotine. AO, L-aspartate oxidase; BBL, berberine bridge enzyme-like protein; MPO, methyleputrescine oxidase; NND, nicotine N-demethylase; PMT, putrecine N-methyltransferase; QPT, quinolinate phosphoribosyltransferase; QS, quinolinate synthase.

The key genes involved in the synthesis of nicotine and nornicotine alkaloids in *Nicotiana *leaves (Figure 6) are listed in Additional file 14 and the corresponding transcripts in root, leaf and flower are shown. The expression data obtained from the hybridization of specific Affymetrix probes (100% match with the targeted sequences) with leaf RNA isolated from *N. sylvestris *and *N. tomentosiformis *provided data similar to FPKM expression, except for four *N. tomentosiformis *genes - *NtomQPT1*, *NtomBBL3*, *NtomNND1 *and *NtomNND2*. However, these four genes were found to be expressed in the leaf of *N. tomentosiformis *plants subjected to RNA-seq analyses. The plants that were used for the RNA-seq analyses were fully mature compared with the young plantlets that were used for the Tobacco Exon Array hybridization, which may indicate that the four genes are more highly expressed in mature leaves than in the primary leaves, suggesting that these genes may possibly affect the alkaloid pathway. Similar to the Cd genes described above, this type of comparison confirms that the design of the Affymetrix exon probes is suitable for the analyses of gene expression in both *N. sylvestris *and *N. tomentosiformis*.

The higher accumulation of nicotine in *N. sylvestris *compared with *N. tomentosiformis *is because of the relatively large deletion that encompasses the NIC2 locus of *N. tomentosiformis*. Therefore, the low-nicotine phenotype is often associated with *nic2 *mutations [72]. In *nic1nic2 *mutant roots, BBL transcripts are strongly reduced [73], attesting that berberine bridge enzyme-like (BBL) genes are regulated by the NIC loci in the roots. Our data confirm that *BBL1 *and *BBL3 *are particularly expressed in the roots of both *Nicotiana *species. However, no large differences in transcript levels were found, possibly suggesting that BBL gene regulation is not as different as suspected between *N. sylvestris *and *N. tomentosiformis*, and the effect of the *nic2 *deletion is apparent somewhere else within the nicotine biosynthesis pathway. In this context, our data show that the expression of a large set of genes involved in nicotine biosynthesis, for example, L-aspartate oxidase (AO), quinolinate synthase (QS), quinolinate phosphoribosyltransferase (QPT), and putrecine N-methyltransferase (PMT), are strongly up-regulated in the roots of *N. sylvestris *compared with *N. tomentosiformis*; indeed, *PMT *expression is not detected in the roots of *N. tomentosiformis*. Four different *PMT *genes have been found in *N. tabacum *[74] and, based on sequence analogy, three of them [75] likely originate from *N. sylvestris *(Additional file 15). Surprisingly, the two copies of *PMT *that are present in *N. tomentosiformis *are similar to only one *PMT *gene in *N. tabacum *(*NtPMT-2*). This finding suggests that because of the lack of the three other PMT copies in *N. tomentosiformis*, the full pathway for nicotine synthesis is certainly different in *N. tomentosiformis *than in *N. sylvestris*, which has three PMT copies that are related to *N. tabacum*, NtPMT-1, -3 and -4 (Additional file 15).

The up-regulation of *PMTs*, *AO *and QS in *N. sylvestris *compared with *N. tomentosiformis *attests that the early steps in the pathway that lead to the synthesis of nicotinic acid are also particularly active in *N. sylvestris *and certainly play a major role in nicotine synthesis. Recent data reported by Shoji and Hashimoto [76] suggest that tobacco MYC2 regulates PMT-2 and QPT-2 by interacting with specific promoter regions. It is therefore tempting to speculate that regulation occurs differently via MYC2 in *N. sylvestris *and *N. tomentosiformis*. Conversely, because AO and QS are located in the plastids and are involved in NAD synthesis from aspartate via quinolinic acid [77], they are likely regulated via nuclear cross-talk that is possibly more active in *N. sylvestris *than in *N. tomentosiformis *(Additional file 14).

In species of the *Nicotiana *genus, the conversion of nicotine to nornicotine, which is the precursor of the tobacco nitrosamine N'-nitrosonornicotine, is mediated by nicotine N-demethylase enzymes (NND) encoded by the CYP82E subfamily of cytochrome P450 genes. Four genes from this gene family are reported to be distributed in the *N. sylvestris *and *N. tomentosiformis *genomes. CYP82E4 is the dominant factor in senescence-inducible nornicotine production, whereas CYP82E5v2 is involved in nicotine conversion in the green leaves; both of them are found in *N. tomentosiformis*, along with CYP82E3. In *N. sylvestris*, one such gene, *CYP82E2*, has been found [78]. Searches in both these *Nicotiana *genomes revealed that *N. sylvestris *has five related genes, and *N. tomentosiformis *has four. A comparison of the phylogenetic trees (Additional file 16) confirms that three of the *N. tomentosiformis *genes are related to the *N. tabacum CYP82E3*, *CYP82E4 *and *CYP82E5 *genes and that one of the *N. sylvestris *genes corresponds to *N. tabacum CYP82E10 *[79]. The data presented in Additional file 14 and Additional file 16 show that *NtomNND-1 *is evolutionarily close to one copy of *CYP82E4*and highly expressed in flowers, whereas its expression in leaves is not supported by Affymetrix data. To our knowledge, the high expression of a nicotine demethylase gene in flowers has not yet been described; the gene product possibly plays a role in protection against insects. Conversely, the *NsylNND-1 *that is evolutionary close to the *N. tabacum CYP82E10 *is highly expressed in roots, confirming the findings of an earlier study [79]. The high expression of the three *N. tomentosiformis *genes related to the *N. tabacum CYP82E3*, *CYP82E4 *and *CYP82E5 *genes suggests that *N. tomentosiformis *is globally a more active producer of nornicotine than *N. sylvestris*, which is the opposite of what was found for nicotine synthesis (see above).

## Conclusions

Draft genomes of *N. sylvestris *and *N. tomentosiformis *were assembled from Illumina short reads; the assemblies cover 83.3% and 71.7% of the calculated genome sizes (2.68 Gb and 2.36 Gb), respectively. Both assemblies have an N50 size of about 80 kb. The repeat content was determined to be 72 to 75% with a higher proportion of retrotransposons and copia-like LTRs in *N. tomentosiformis *compared with *N*. *sylvestris*. The reported draft genomes offer good coverage of coding regions, as exemplified by the heavy-metal transport and alkaloid metabolism analyses. The examination of the terpenoid metabolism gene families is more challenging because their members are numerous and highly similar, and will require further investigations.

Tobacco SSR markers were mapped to both assemblies and a 65% concordance with PCR amplification data reported previously [13] was obtained. In addition, 5 to 7% of the markers that amplified in only one of the species could actually be mapped in both. Of the markers on the *N. acuminata *and *N. tomentosiformis *genetic maps, 74 to 78% could be mapped to the genome assemblies.

The COSII markers from these two genetic maps were also mapped to both assemblies. In this case, only 31 to 34% of them could be mapped onto the *N. sylvestris *and *N. tomentosiformis *assemblies, although when the same method was applied on the tomato genome, 84% of the markers present on the tomato genetic map could be mapped. This discrepancy could be due either to the still relatively high fragmentation of the *Nicotiana *genome assemblies, or to the COSII PCR primers not being suitable for the *Nicotiana *species.

The transcriptome assemblies revealed the expression of 44,000 to 53,000 transcripts in roots, leaves or flowers. Flowers had the most expressed transcripts, with about 3,500 expressed transcripts not detectable in roots or leaves. The merged species transcriptomes yielded 66,000 to 68,000 expressed transcripts, encoding 39,000 proteins. When these transcripts were clustered with genes from tomato and *Arabidopsis*, a core set of about 7,100 clusters, a Solanaceae-specific set of about 2,800 clusters, and a *Nicotiana*-specific set of about 3,600 clusters were identified.

Phenotypic differences observed between *N. sylvestris *and *N. tomentosiformis *could be explained by investigating the number of genes for specific protein families of the three metabolic pathways and their expressions in root, leaf and flower.

The SOL100 initiative aims to sequence a wide range of Solanaceae species to deepen our understanding of this plant family and improve breeding of its cultivars. The draft genomes of *N. sylvestris *and *N. tomentosiformis *represent a significant contribution to this effort. Both are the ancestral species of allotetraploid tobacco (*N. tabacum*) with a 4.5 Gb genome, which currently represents a formidable challenge due to its high complexity. The genomes of the ancestor species provide a significant advance towards the assembly of the *N. tabacum *genome and illustrate a general strategy for the genomes of other polyploidy species such as wheat and cotton. These new genomes will increase the value of the already existing Solanaceae resources by providing additional comparative information at the genome and transcriptome levels and will help improve our understanding of plant metabolism and evolution.

## Materials and methods

### Illumina sequencing

Young leaves, roots and flowers of *N. sylvestris *(USNGC TW136, PI555569) and *N. tomentosiformis *(USNGC TW142, PI555572) grown in a greenhouse were collected. DNA extraction was performed using Qiagen DNAeasy Plant Maxi Kit (Qiagen, Düsseldorf, Germany) from fresh leaves. RNA extraction was performed using the Qiagen RNAeasy Mini Kit (Qiagen).

Short insert 'paired-end' libraries were prepared using the Illumina TruSeq DNA Sample Preparation Kit version 2 according to the manufacturer's instructions, or with few modifications if prepared by Fasteris. For Fasteris, 2.1 mg of genomic DNA was broken using BioRuptor (Diagenode, Liège, Belgium); ends were repaired using Klenow and polynucleotide kinase, and then Fasteris-modified adapters were ligated to the inserts. After size selection on agarose gel, the libraries were amplified by ten PCR cycles, and then purified and quantified.

Long insert 'mate-pair' libraries were prepared using the Illumina Mate Pair Library Prep Kit version 2 according to the manufacturer's instructions, or using a Fasteris-developed protocol in which 10 mg of genomic DNA were broken into fragments of approximately 2 to 5 kb using Covaris (KBioSciences, Herts, UK) and purified on 0.7% agarose gel to recover fragments of 3 kb and 5 kb. After end repair, a Fasteris-designed spacer was ligated and the fragments were circularized. Non-circular fragments were eliminated and then the DNA was broken using Covaris to generate fragments of 400 bp, which were end repaired, ligated with Illumina adapters, purified on agarose gel and amplified by PCR for 12 cycles.

RNA-seq libraries were constructed using Illumina's TruSeq RNA Sample prep Kit protocol according to the manufacturer's instructions. All the libraries (short insert 'paired-end', long insert 'mate-pair', and RNA-seq) were sequenced on an Illumina HiSeq-2000 using version 3 chemistry and flow-cells with runs of 2 × 100 bases. Base calling and sample demultiplexing were performed using Illumina's HiSeq Control Software and the CASAVA pipeline. The data for the *N. sylvestris *and *N. tomentosiformis *RNA-seq triplicates have been uploaded to the EBI Sequence Read Archive under accession numbers ERP002501 and ERP002502, respectively.

### Genome size estimation

We estimated the genome size of *N. sylvestris *and *N. tomentosiformis *using the 31-mer depth distribution of all the non-overlapping paired-end libraries, as described previously [36,50-52]. Briefly, the genome size is obtained by dividing the total number of 31-mers considered to be error-free by their most frequent depth of coverage.

### Genome assembly

The raw DNA reads from *N. sylvestris *and *N. tomentosiformis *were preprocessed by first trimming 3' bases with qualities lower than 30, and then discarding reads shorter than 50 bases or with less than 90% of the bases with qualities lower than 30. The paired-end libraries with insert sizes shorter than 200 bases were further preprocessed using FLASH [80] to merge the paired-end reads into extended single reads.

The paired and single reads from the paired-end libraries were then assembled into contigs using SOAPdenovo [81] with a k-mer of 63, and the paired reads from paired-end and mate-pair libraries were used for scaffolding by increasing library size. To improve scaffolding, mate-pair libraries from closely related *Nicotiana *species were also used. Gaps that resulted from the scaffolding were closed using GapCloser and all sequences shorter than 200 bases were discarded from the final assemblies.

Superscaffolding using the tobacco WGP™ physical map was possible because it is based on sequencing tags, and the origin of the WGP contigs have been annotated. Briefly, WGP tags of S or T origin were mapped to the *N. sylvestris *or *N. tomentosiformis *sequences, respectively. Superscaffolds were created when two or more sequences could be anchored and oriented unambiguously to a WGP contig. The *N. sylvestris *and *N. tomentosiformis *genome assemblies have been submitted to GenBank BioProjects PRJNA182500 and PRJNA182501, respectively. The *N. sylvestris *whole genome shotgun project has been deposited at DDBJ/EMBL/GenBank under the accession ASAF00000000. The version described in this paper is version ASAF01000000. The *N. tomentosiformis *whole genome shotgun project has been deposited at DDBJ/EMBL/GenBank under the accession ASAG00000000. The version described in this paper is version ASAG01000000. The raw sequencing data used for the assemblies of *N. sylvestris *and *N. tomentosiformis *genomes have been submitted to the EBI Sequence Read Archive under accession numbers ERP002501 and ERP002502.

### Repeat content estimation

The repeat content of the *N. sylvestris *and *N. tomentosiformis *genome assemblies were estimated using RepeatMasker [82] with the eudicot repeat library available from the Sol Genomics Network, the TIGR Solanaceae repeat library, and RepeatScout [83] libraries created using sequences of at least 200 kb from the draft genome assemblies of *N. sylvestris *and *N. tomentosiformis*. Classification of the repeat types was done using the NCBI BLASTN [84-86] hits to known repeat elements.

### Genetic markers

PCR primers for the SSR markers have been reported previously [13] and the COSII makers from Sol Genomics Network were mapped to the draft assembly genomes of *N. sylvestris *and *N. tomentosiformis *using LAST [87]. Only the primer pairs that could be mapped with at least 95% identity and that yielded a unique PCR product were retained.

### Pathway gene identification and quantification

Genomic regions containing genes that potentially encode proteins from the selected pathways were identified by mapping homologous proteins from other species to the genome assemblies using BLAT [88] and manually curating the hits. Probes from the Tobacco Exon Array [49] were selected by mapping them to the identified genome regions using LAST [87] and retaining only perfect matches that could be mapped uniquely. Quantification of gene expression was obtained by summing the Cufflinks [89] FPKM values of the transcripts that overlapped the identified genome regions.

### *De novo *transcriptome assembly

All the reads were preprocessed to clip the overrepresented sequences reported by FastQC [90]. After clipping, the 3' ends of the reads were quality trimmed with a quality threshold of 20 and artifacts were removed. Finally, reads of at least 50 nucleotides with at least 75% nucleotides of quality 20 or more were kept. The clipping, trimming and filtering were performed using the fastx toolkit [91]. Transcripts were assembled using the Trinity *de novo *assembly pipeline [92]; the peptide prediction program contained within this software suite was used to predict peptides from the assembled transcripts.

Transcriptome assembly was performed using the 'Tuxedo' suite of tools. Reads were mapped to the appropriate genome assembly using the Bowtie2/Tophat2 [93,94] pipeline with the default parameters. Transcript generation was performed using the Cufflinks tools [89] and merged using Cuffmerge. A representative set of transcript sequences was generated using the 'gtf_to_fasta' component of Cufflinks.

### Transcript and protein quality

The ORF finding utility included in the Trinity software package (version January 2012) was used to find ORFs in the inferred transcripts. Candidate peptide sequences were culled at a minimum length of 100 amino acids. The search for sequences homologous to the ORFs was performed using BLAST [84], with the UniProt Knowledgebase and the Swiss-Prot subset as reference databases. A reasonably stringent e-value cutoff of 1E-30 was used and only one hit was retained for each sequence. To determine which of the best hits were mutual, a reverse search (reference database against transcripts) was also performed using the same parameters.

### Functional comparison to other species

Orthologous and paralogous genes between our sequences and those from other species were clustered using OrthoMCL [54,55]. To ensure comparability, we used the same ORF finding software on the *Arabidopsis *(TAIR10 sequence) and tomato sequences (ITAG version 2.3) to derive peptide sequences and then used only sequences of 100 amino acids or longer. An all-against-all sequence search was performed using BLAST [84] (version 2.2.23+) with default parameters and the results of this search were used as the input to OrthoMCL, which was run using the default parameter set (percentMatchCutoff = 50; e-valueExponentCutoff = -5). The OrthoMCL protein group output files were further processed using in-house Python scripts, and visualized in R [95] as a Venn diagram using the CRAN package 'VennDiagram' [96].

### Functional annotation

The EFICAz^2.5 ^software [97-99] was used to predict EC numbers for the protein sequences predicted from the transcripts of the pooled tissue samples. The InterProScan software [100], version 4.8 was used to assign GO terms to the protein sequences.

## Abbreviations

AO: L-aspartate oxidase; BBL: berberine bridge enzyme-like; CAX: cation proton exchanger; COS: conserved ortholog set; EC: Enzyme Commission; EST: expressed sequence tag; FPKM: fragments per kilobase of transcript per million mapped reads; GO: Gene Ontology; HMA: heavy metal ATPase; LTR: long terminal repeat; MRP: multidrug resistance-associated protein; NND: nicotine N-demethylase; NS: nicotianamine synthetase; ORF: open reading frame; PCS: phytochelatin synthase; PMT: putrecine N-methyltransferase; QPT: quinolinate phosphoribosyltransferase; QS: quinolinate synthase; SNP: single nucleotide polymorphism; SSR: simple sequence repeat; WGP: Whole Genome Profiling.

## Competing interests

The research described in this article was financially supported by Philip Morris International. The objective of this work was to provide a contribution to the SOL100 initiative and was not for the development of tobacco-based products.

## Authors' contributions

NS, NVI and MCP conceived and designed the study. SG and LB provided plant material and contributed to the biological interpretation of the results. NB provided the genetic maps of *Nicotiana sylvestris *and *Nicotiana tomentosiformis*. SO prepared the libraries and performed the Illumina sequencing. NS and JB carried out genome and transcriptome analysis. NS, JB, LB, and NVI wrote the manuscript. NVI supervised the study. All authors read and approved the final manuscript.

## Supplementary Material

Additional file 1 Statistics of the *Nicotiana sylvestris *sequencing libraries. Click here for file

Additional file 2 Statistics of the *Nicotiana tomentosiformis *sequencing libraries. Click here for file

Additional file 3 Repetitive elements in the *Nicotiana sylvestris *and *Nicotiana tomentosiformis *genomes. Click here for file

Additional file 4 **Comparisons between the SSR mapping to the draft genomes and the PCR amplification test results**. Click here for file

Additional file 5 Genetic map of *Nicotiana acuminata*. Click here for file

Additional file 6 **Comparisons between the SSR mapping to the draft genomes and existing genetic maps**. Click here for file

Additional file 7 Genetic map of *Nicotiana tomentosiformis*. Click here for file

Additional file 8 **Comparisons between the COSII mapping to the draft genomes and existing genetic maps**. Click here for file

Additional file 9 Statistics of the *Nicotiana sylvestris *and *Nicotiana tomentosiformis *RNA-seq libraries. Click here for file

Additional file 10 **Transcriptome sequence number and length distribution**. Click here for file

Additional file 11 Genomic GO term (Biological Process) enrichment in *Nicotiana sylvestris *and *Nicotiana tomentosiformis *genes. Click here for file

Additional file 12 Non-exhaustive list of gene copies potentially involved in cadmium/zinc (Cd/Zn) accumulation in *Nicotiana *leaves. Click here for file

Additional file 13 Phylogenetic tree of CAX proteins from the *N. sylvestris*, *N. tomentosiformis *and Arabidopsis genomes. The *N. sylvestris *and *N. tomentosiformis *proteins are numbered according to the rows of Additional file 12. Bootstrap percentages are shown at each node. Click here for file

Additional file 14 Key genes involved in the synthesis of nicotine and nornicotine alkaloids in *Nicotiana *leaves. Click here for file

Additional file 15 Phylogenetic tree of PMT proteins from the *N. sylvestris*, *N. tomentosiformis *and *N. tabacum *genomes. The *N. sylvestris *and *N. tomentosiformis *proteins are numbered according to the rows of Additional file 14. Bootstrap percentages are shown at each node. Click here for file

Additional file 16 Phylogenetic tree of CYP82E cytochrome P450 proteins and orthologs from the *N. sylvestris *and *N. tomentosiformis *genomes. The *N. sylvestris *and *N. tomentosiformis *proteins are numbered according to the rows of Additional file 14. Bootstrap percentages are shown at each node. Click here for file
