# Supplementary material for: Reference genomes and transcriptomes of Nicotiana sylvestris and Nicotiana tomentosiformis
Source: Genome Biol. 2013 Jun 17;14(6):R60. doi: 10.1186/gb-2013-14-6-r60 (PMC3707018; doi:10.1186/gb-2013-14-6-r60)
Supplement: Additional file 1 — Statistics of the Nicotiana sylvestris sequencing libraries. [file gb-2013-14-6-r60-S1.DOCX]

Additional file 1: Statistics of the *Nicotiana sylvestris* sequencing libraries.

| Library type | Read size (bp) | Insert size | Cleaned reads | Expected coverage |
| --- | --- | --- | --- | --- |
| Paired-end | 2×100 | 180 bases | 1,249,808,412 | 47.5× |
| Paired-end | 2x100 | 300 bases | 1,057,102,557 | 38.6× |
| Paired-end | 2×100 | 1 kb | 42,216,128 | 1.6× |
| Mate-pair | 2×100 | 3 kb | 98,524,837 | 2.8× |
| Mate-pair | 2×100 | 4 kb | 63,727,279 | 1.8× |
| Mate-pair | 2×100 | 4 kb | 51,368,983 | 1.5× |
