# Supplementary material for: Reference genomes and transcriptomes of Nicotiana sylvestris and Nicotiana tomentosiformis
Source: Genome Biol. 2013 Jun 17;14(6):R60. doi: 10.1186/gb-2013-14-6-r60 (PMC3707018; doi:10.1186/gb-2013-14-6-r60)
Supplement: Additional file 2 — Statistics of the Nicotiana tomentosiformis sequencing libraries. [file gb-2013-14-6-r60-S2.DOCX]

Additional file 2: Statistics of the *Nicotiana tomentosiformis* sequencing libraries.

| Library type | Read size (bp) | Insert size | Cleaned reads | Expected coverage |
| --- | --- | --- | --- | --- |
| Paired-end | 2×100 | 140 bases | 1,730,522,445 | 65.7× |
| Paired-end | 2×100 | 175 bases | 823,913,833 | 31.0× |
| Paired-end | 2×100 | 350 bases | 804,501,117 | 30.2× |
| Paired-end | 2×100 | 385 bases | 462,732,217 | 17.6× |
| Paired-end | 2×100 | 1 kb | 34,860,106 | 1.3× |
| Mate-pair | 2×100 | 3 kb | 8,065,420 | 0.25× |
| Mate-pair | 2×100 | 5 kb | 7,750,383 | 0.25× |
