# Supplementary material for: Reference genomes and transcriptomes of Nicotiana sylvestris and Nicotiana tomentosiformis
Source: Genome Biol. 2013 Jun 17;14(6):R60. doi: 10.1186/gb-2013-14-6-r60 (PMC3707018; doi:10.1186/gb-2013-14-6-r60)
Supplement: Additional file 3 — Repetitive elements in the Nicotiana sylvestris and Nicotiana tomentosiformis genomes. [file gb-2013-14-6-r60-S3.DOCX]

Additional file 3: Repetitive elements in the *Nicotiana sylvestris* and *Nicotiana tomentosiformis* genomes. The repeat contents were estimated using RepeatMasker with the eudicot repeat library available from the Sol Genomics Network, the TIGR Solanaceae repeat library, and de novo RepeatScout libraries.

|  | ***N. sylvestris*** | | ***N. tomentosiformis*** | |
| --- | --- | --- | --- | --- |
| **Repeat elements** | **Bases** | **% all** | **Bases** | **% all** |
| BASES_MASKED | 1605541978 | 71.95 | 1266206541 | 74.84 |
| BASES_N | 182258661 | 8.17 | 47655588 | 2.82 |
| DNA/En-Spm | 16928 | 0 | 14272 | 0 |
| DNA/En-Spm-nsyl | 2577438 | 0.12 | 962238 | 0.06 |
| DNA/En-Spm-ntom | 631391 | 0.03 | 1451899 | 0.09 |
| DNA/Harbinger-nsyl | 559838 | 0.03 | 157311 | 0.01 |
| DNA/Harbinger-ntom | 425716 | 0.02 | 1183737 | 0.07 |
| DNA/hAT-nsyl | 2865626 | 0.13 | 663748 | 0.04 |
| DNA/hAT-ntom | 2067013 | 0.09 | 3355505 | 0.2 |
| DNA/Mite-nsyl | 1011996 | 0.05 | 11268 | 0 |
| DNA/Mite-ntom | 210350 | 0.01 | 795160 | 0.05 |
| DNA/MuDR-nsyl | 977474 | 0.04 | 267962 | 0.02 |
| DNA/MuDR-ntom | 840098 | 0.04 | 2151110 | 0.13 |
| DNA/NicCL3 | 1259 | 0 | 6172 | 0 |
| DNA/NicCL3-nsyl | 217271 | 0.01 | 487583 | 0.03 |
| DNA/NicCL3-ntom | 2868063 | 0.13 | 12112445 | 0.72 |
| DNA/NicCL7_30 | 408941 | 0.02 | 1522054 | 0.09 |
| DNA/NicCL7_30-nsyl | 42082212 | 1.89 | 16777267 | 0.99 |
| DNA/NicCL7_30-ntom | 23040894 | 1.03 | 75991403 | 4.49 |
| DNA-nsyl | 539419 | 0.02 | 85323 | 0.01 |
| DNA-ntom | 1417248 | 0.06 | 2507492 | 0.15 |
| DNA/ribosomal | 18411 | 0 | 22718 | 0 |
| DNA/ribosomal-nsyl | 2252 | 0 | 2932 | 0 |
| DNA/Satellite | 84 | 0 | 339 | 0 |
| DNA/TcMar-nsyl | 3256200 | 0.15 | 18045 | 0 |
| DNA/TcMar-ntom | 276518 | 0.01 | 1308012 | 0.08 |
| DNA/TcMar-Pogo-nsyl | 21359 | 0 | 476 | 0 |
| DNA/Telomere | 4239 | 0 | 5573 | 0 |
| DNA/Telomere-nsyl | 321809 | 0.01 | 110717 | 0.01 |
| DNA/Telomere-ntom | 117370 | 0.01 | 210825 | 0.01 |
| LINE | 399717 | 0.02 | 357716 | 0.02 |
| LINE-nsyl | 5073591 | 0.23 | 2015974 | 0.12 |
| LINE-ntom | 355671 | 0.02 | 460484 | 0.03 |
| Low_complexity | 10145060 | 0.45 | 9723109 | 0.57 |
| LTR/Copia | 2643789 | 0.12 | 2044782 | 0.12 |
| LTR/Copia-nsyl | 70292366 | 3.15 | 15807616 | 0.93 |
| LTR/Copia-ntom | 34263968 | 1.54 | 79716005 | 4.71 |
| LTR/Copia-retrolyc1 | 834 | 0 | 485 | 0 |
| LTR/Copia-retrolyc1-nsyl | 204247 | 0.01 | 35131 | 0 |
| LTR/Copia-Tnt1 | 1317465 | 0.06 | 2001056 | 0.12 |
| LTR/Copia-Tnt1-nsyl | 54348983 | 2.44 | 8505067 | 0.5 |
| LTR/Copia-Tnt1-ntom | 32993735 | 1.48 | 114525292 | 6.77 |
| LTR/Copia-Tnt2 | 8162 | 0 | 471 | 0 |
| LTR/Copia-Tnt2-nsyl | 1843977 | 0.08 | 583031 | 0.03 |
| LTR/Copia-Tto1 | 54853 | 0 | 4554 | 0 |
| LTR/Copia-Tto2 | 166 | 0 | 389 | 0 |
| LTR/Copia-Tto2-nsyl | 3614044 | 0.16 | 1882851 | 0.11 |
| LTR/Copia-Tto2-ntom | 2005992 | 0.09 | 2384357 | 0.14 |
| LTR/Gypsy | 11170 | 0 | 11642 | 0 |
| LTR/Gypsy-nsyl | 386764199 | 17.33 | 105525720 | 6.24 |
| LTR/Gypsy-ntom | 76294797 | 3.42 | 238247258 | 14.08 |
| LTR-nsyl | 161879145 | 7.25 | 20708314 | 1.22 |
| LTR-ntom | 23002062 | 1.03 | 69457892 | 4.11 |
| RC/Helitron-nsyl | 1411960 | 0.06 | 219580 | 0.01 |
| RC/Helitron-ntom | 846432 | 0.04 | 1281799 | 0.08 |
| Retroelement-nsyl | 263059 | 0.01 | 185619 | 0.01 |
| retroposon/Au | 251 | 0 | 402 | 0 |
| retrotransposon | 12192 | 0 | 13230 | 0 |
| retrotransposon-nsyl | 182390040 | 8.17 | 59525590 | 3.52 |
| retrotransposon-ntom | 48250834 | 2.16 | 161188425 | 9.53 |
| rRNA | 6817 | 0 | 11069 | 0 |
| rRNA-nsyl | 4374043 | 0.2 | 175832 | 0.01 |
| rRNA-ntom | 1563966 | 0.07 | 6127688 | 0.36 |
| Satellite | 0 | 0 | 320 | 0 |
| Simple_repeat | 4954900 | 0.22 | 4809855 | 0.28 |
| SINE | 3314 | 0 | 4441 | 0 |
| SINE-nsyl | 973946 | 0.04 | 170820 | 0.01 |
| SINE-ntom | 3062878 | 0.14 | 5068908 | 0.3 |
| transposon | 822816 | 0.04 | 629718 | 0.04 |
| transposon-nsyl | 26068866 | 1.17 | 6424936 | 0.38 |
| transposon-ntom | 6730213 | 0.3 | 15538350 | 0.92 |
| Unknown | 19374 | 0 | 30643 | 0 |
| Unknown/Nsyl-nsyl | 168661039 | 7.56 | 12165228 | 0.72 |
| Unknown/Ntom-ntom | 29112026 | 1.3 | 103931768 | 6.14 |
