# Supplementary material for: Reference genomes and transcriptomes of Nicotiana sylvestris and Nicotiana tomentosiformis
Source: Genome Biol. 2013 Jun 17;14(6):R60. doi: 10.1186/gb-2013-14-6-r60 (PMC3707018; doi:10.1186/gb-2013-14-6-r60)
Supplement: Additional file 4 — Comparisons between the SSR mapping to the draft genomes and the PCR amplification test results. [file gb-2013-14-6-r60-S4.DOCX]

Additional file 4: Comparisons between the SSR mapping to the draft genomes and the PCR amplification test results.

|  | Assemblies | | | |
| --- | --- | --- | --- | --- |
| Amplification | *N. sylvestris* | *N. tomentosiformis* | *N. sylvestris* and  *N. tomentosiformis* | None |
| *N. sylvestris* | 531 (65%) | 24 (3%) | 57 (7%) | 207 (25%) |
| *N. tomentosiformis* | 18 (4%) | 305 (65%) | 70 (15%) | 78 (17%) |
| *N. sylvestris* and  *N. tomentosiformis* | 129 (16%) | 345 (44%) | 81 (10%) | 231 (29%) |
| Unknown | 88 (31%) | 81 (28%) | 26 (9%) | 92 (32%) |
