# Supplementary material for: Reference genomes and transcriptomes of Nicotiana sylvestris and Nicotiana tomentosiformis
Source: Genome Biol. 2013 Jun 17;14(6):R60. doi: 10.1186/gb-2013-14-6-r60 (PMC3707018; doi:10.1186/gb-2013-14-6-r60)
Supplement: Additional file 5 — Genetic map of Nicotiana acuminata. [file gb-2013-14-6-r60-S5.DOCX]

Additional file 5: Genetic map of *Nicotiana acuminata*.

| **Marker** | **LG** | **Position** |
| --- | --- | --- |
| C2At2g01110 | 1 | 0 |
| C2At3g50860 | 1 | 7.2 |
| PT51914 | 1 | 13.6 |
| C2At2g14835 | 1 | 16.4 |
| C2At2g38730 | 1 | 26.4 |
| C2At1g54520 | 1 | 32.5 |
| PT50294 | 1 | 32.5 |
| C2At1g09070 | 1 | 37.6 |
| C2At1g56500 | 1 | 42.1 |
| C2At1g25260 | 1 | 50.7 |
| C2At1g02150 | 1 | 52.7 |
| C2At3g52120 | 1 | 62.3 |
| C2At3g08030 | 1 | 64.4 |
| PT53728 | 1 | 74.2 |
| C2At1g03360 | 1 | 77.9 |
| C2At4g21710 | 2 | 0 |
| C2At1g11430 | 2 | 4.8 |
| C2At1g65900 | 2 | 9.6 |
| PT53753 | 2 | 9.6 |
| PT52128 | 2 | 13.2 |
| C2At3g01180 | 2 | 16.8 |
| C2At2g04700 | 2 | 18.3 |
| C2At3g55250 | 2 | 19.8 |
| C2At3g27200 | 2 | 23.8 |
| C2At5g45950 | 2 | 28.9 |
| C2At3g28670 | 2 | 31.9 |
| PT51924 | 2 | 36.6 |
| PT50051 | 2 | 38.4 |
| C2At2g34560 | 2 | 40.1 |
| PT52117 | 2 | 42.9 |
| PT53847 | 2 | 42.9 |
| C2At4g20070 | 2 | 48.4 |
| C2At5g66530 | 2 | 55.6 |
| PT50178 | 2 | 55.6 |
| PT52119 | 2 | 57.8 |
| PT60903 | 2 | 58.9 |
| PT52734 | 2 | 62.6 |
| C2At5g67370 | 2 | 66.5 |
| C2At4g33985 | 2 | 70.4 |
| C2At4g34090 | 2 | 70.4 |
| PT53810 | 2 | 86.4 |
| C2At1g30540 | 2 | 106.3 |
| C2At3g46780 | 2 | 106.3 |
| PT53756 | 2 | 106.3 |
| PT53971 | 2 | 106.3 |
| PT54024 | 2 | 106.3 |
| PT50313 | 3 | 0 |
| C2At1g64770 | 3 | 10.2 |
| C2At1g27600 | 3 | 19.1 |
| C2At1g29320 | 3 | 19.1 |
| C2At2g26210 | 3 | 23.2 |
| C2At1g07310 | 3 | 27.2 |
| PT51852 | 3 | 30.9 |
| C2At5g51110 | 3 | 34.6 |
| C2At1g51160 | 3 | 45.7 |
| PT50175 | 3 | 47.8 |
| C2At1g79600 | 3 | 53.2 |
| PT50055 | 3 | 57.4 |
| C2At3g48610 | 3 | 62.4 |
| C2At1g74730 | 3 | 65 |
| C2At1g74470 | 3 | 69.7 |
| C2At1g80170 | 3 | 74.4 |
| C2At3g14200 | 3 | 80 |
| C2At3g14075 | 3 | 83.4 |
| C2At3g13700 | 3 | 85.1 |
| C2At3g18270 | 3 | 98.1 |
| C2At1g68100 | 4 | 0 |
| C2At3g19895 | 4 | 6.5 |
| C2At3g17040 | 4 | 12.9 |
| C2At2g39580 | 4 | 14.2 |
| C2At4g25650 | 4 | 15.5 |
| C2At5g37290 | 4 | 16.8 |
| PT50433 | 4 | 20.5 |
| PT53979 | 4 | 20.5 |
| PT54818 | 4 | 20.5 |
| PT50331 | 4 | 24.2 |
| PT52092 | 4 | 28.1 |
| PT50345 | 4 | 30.1 |
| C2At1g71810 | 4 | 37.8 |
| C2At5g10920 | 4 | 50.5 |
| C2At1g46480 | 4 | 58.8 |
| PT50277 | 4 | 60.9 |
| PT53721 | 4 | 62.9 |
| PT50334 | 4 | 68.9 |
| C2At1g20575 | 4 | 74.8 |
| C2At3g55800 | 5 | 0 |
| C2At4g18593 | 5 | 0 |
| C2At5g05270 | 5 | 0 |
| C2At5g49510 | 5 | 0 |
| C2At5g09380 | 5 | 6.5 |
| C2At5g23520 | 5 | 13 |
| PT50316 | 5 | 29.8 |
| C2At2g28880 | 5 | 46.8 |
| C2At5g37360 | 5 | 50.8 |
| PT52027 | 5 | 57.8 |
| PT50036 | 5 | 65 |
| PT60465 | 5 | 69 |
| C2At3g54840 | 5 | 71 |
| C2At5g14520 | 5 | 73 |
| C2At5g16710 | 5 | 75 |
| PT52034 | 5 | 75 |
| PT50195 | 5 | 78.3 |
| C2At2g25740 | 6 | 3.8 |
| C2At2g32950 | 6 | 7.3 |
| C2At4g11120 | 6 | 15.7 |
| PT52083 | 6 | 23.3 |
| PT50113 | 6 | 29.8 |
| PT50003 | 6 | 38.4 |
| C2At5g62530 | 6 | 41.7 |
| C2At1g18640 | 6 | 44.9 |
| C2At2g43360 | 6 | 58.1 |
| C2At4g34215 | 6 | 58.1 |
| C2At1g44760 | 6 | 65.3 |
| C2At1g03150 | 6 | 80.8 |
| PT51946 | 6 | 86.5 |
| C2At1g52220 | 6 | 91.9 |
| C2At1g20050 | 6 | 97.3 |
| C2At3g63200 | 6 | 97.3 |
| C2At1g16870 | 6 | 102.8 |
| PT51907 | 7 | 0 |
| C2At4g26680 | 7 | 4.4 |
| PT53995 | 7 | 5.6 |
| PT53836 | 7 | 6.7 |
| PT52139 | 7 | 11.5 |
| C2At1g78620 | 7 | 22.8 |
| C2At3g15290 | 7 | 32.6 |
| PT51957 | 7 | 34.9 |
| C2At1g04970 | 7 | 42 |
| C2At4g12740 | 7 | 57.3 |
| PT52100 | 7 | 63.3 |
| C2At2g37500 | 7 | 69.7 |
| C2At2g37025 | 7 | 76 |
| C2At3g09920 | 7 | 81 |
| C2At3g15410 | 8 | 0 |
| C2At2g20860 | 8 | 6.4 |
| C2At4g26750 | 8 | 6.4 |
| PT50042 | 8 | 6.4 |
| C2At2g46370 | 8 | 21.9 |
| C2At4g32770 | 8 | 53.2 |
| C2At5g11450 | 8 | 63.5 |
| C2At5g25630 | 8 | 70 |
| PT53773 | 8 | 76.5 |
| C2At1g62780 | 8 | 83 |
| PT51839 | 8 | 93.4 |
| PT54887 | 8 | 93.4 |
| PT60177 | 8 | 103.7 |
| C2At5g06360 | 9 | 0 |
| C2At3g24160 | 9 | 8.3 |
| C2At5g42740 | 9 | 11.7 |
| C2At3g06790 | 9 | 15.1 |
| C2At5g18580 | 9 | 18.5 |
| C2At3g23590 | 9 | 21.8 |
| PT52140 | 9 | 26.1 |
| C2At1g04530 | 9 | 34.7 |
| PT53798 | 9 | 43.3 |
| C2At4g35930 | 9 | 50.5 |
| C2At1g61150 | 9 | 57.6 |
| C2At4g04930 | 9 | 61.5 |
| cLPT5E7 | 9 | 65.3 |
| PT51862 | 9 | 65.3 |
| PT53988 | 9 | 65.3 |
| C2At5g36210 | 10 | 0 |
| C2At3g51840 | 10 | 11.8 |
| PT50076 | 10 | 22.8 |
| PT53981 | 10 | 22.8 |
| C2At5g04200 | 10 | 25.4 |
| PT50026 | 10 | 32.2 |
| PT50054 | 10 | 41 |
| PT52130 | 10 | 54.9 |
| PT51911 | 10 | 66.2 |
| C2At4g31130 | 10 | 67.7 |
| C2At2g24580 | 10 | 69.2 |
| C2At1g31410 | 10 | 70.7 |
| C2At2g24270 | 11 | 3.1 |
| C2At5g34850 | 11 | 13.4 |
| PT53860 | 11 | 16.2 |
| C2At5g06430 | 11 | 21.9 |
| PT53719 | 11 | 21.9 |
| PT50179 | 11 | 29.6 |
| C2At1g56050 | 11 | 31.6 |
| C2At1g73177 | 11 | 35.1 |
| C2At1g26940 | 11 | 42.1 |
| PT51893 | 11 | 46.9 |
| C2At1g30825 | 11 | 51.6 |
| C2At2g28250 | 11 | 56.7 |
| C2At2g28800 | 11 | 61.8 |
| C2At3g22660 | 11 | 70.7 |
| C2At3g12685 | 11 | 91 |
| C2At5g53000 | 12 | 0 |
| PT50356 | 12 | 2.5 |
| C2At5g21170 | 12 | 5 |
| C2At3g16290 | 12 | 12.6 |
| PT51917 | 12 | 20.4 |
| C2At1g09920 | 12 | 34.3 |
| C2At4g33360 | 12 | 37.6 |
| C2At4g29735 | 12 | 42.6 |
| C2At4g31150 | 12 | 44.6 |
| C2At4g31410 | 12 | 46.6 |
| C2At4g24750 | 12 | 50.6 |
| PT52047 | 12 | 50.6 |
| PT53750 | 12 | 54.6 |
| C2At5g14320 | 12 | 57.9 |
| PT50299 | 12 | 57.9 |
| C2At1g26520 | 12 | 65.8 |
| PT50215 | 12 | 65.8 |
| C2At1g25580 | 12 | 73.5 |
