# Supplementary material for: Reference genomes and transcriptomes of Nicotiana sylvestris and Nicotiana tomentosiformis
Source: Genome Biol. 2013 Jun 17;14(6):R60. doi: 10.1186/gb-2013-14-6-r60 (PMC3707018; doi:10.1186/gb-2013-14-6-r60)
Supplement: Additional file 6 — Comparisons between the SSR mapping to the draft genomes and existing genetic maps. [file gb-2013-14-6-r60-S6.DOCX]

Additional file 6: Comparisons between the SSR mapping to the draft genomes and existing genetic maps.

|  | Assemblies | | | |
| --- | --- | --- | --- | --- |
| Genetic map | *N. sylvestris* | *N. tomentosiformis* | *N. sylvestris* and  *N. tomentosiformis* | None |
| *N. acuminata* | 75 (44%) | 21 (12%) | 50 (30%) | 23 (14%) |
| *N. tomentosiformis* | 5 (2%) | 141 (65%) | 29 (13%) | 42 (19%) |
| *N. acuminata* and *N. tomentosiformis* | 1 (25%) | 1 (25%) | 2 (50%) | 0 (0%) |
| Unknown | 706 (35%) | 605 (30%) | 174 (9%) | 561 (27%) |
