# Supplementary material for: Reference genomes and transcriptomes of Nicotiana sylvestris and Nicotiana tomentosiformis
Source: Genome Biol. 2013 Jun 17;14(6):R60. doi: 10.1186/gb-2013-14-6-r60 (PMC3707018; doi:10.1186/gb-2013-14-6-r60)
Supplement: Additional file 7 — Genetic map of Nicotiana tomentosiformis. [file gb-2013-14-6-r60-S7.DOCX]

Additional file 7: Genetic map of *Nicotiana tomentosiformis*.

| **Marker** | **LG** | **Position** |
| --- | --- | --- |
| C2At5g13450 | 1 | 0 |
| C2At3g52120 | 1 | 5 |
| C2At5g08430 | 1 | 10 |
| C2At1g02150 | 1 | 15 |
| PT53353 | 1 | 20 |
| C2At1g25260 | 1 | 25.5 |
| C2At1g33270 | 1 | 29.1 |
| PT53143 | 1 | 32.6 |
| C2At1g10240 | 1 | 34.9 |
| PT55106 | 1 | 36.7 |
| C2At5g02270 | 1 | 41.1 |
| C2At1g56500 | 1 | 45.4 |
| C2At5g03905 | 1 | 46.8 |
| PT50861 | 1 | 46.8 |
| C2At1g09070 | 1 | 48.2 |
| PT50819 | 1 | 48.2 |
| C2At4g00740 | 1 | 48.5 |
| C2At4g01210 | 1 | 48.8 |
| C2At3g19230 | 1 | 49.1 |
| C2At1g54520 | 1 | 49.4 |
| C2At2g45600 | 1 | 49.4 |
| C2At4g32280 | 1 | 49.4 |
| PT50500 | 1 | 49.4 |
| PT52749 | 1 | 49.4 |
| PT54911 | 1 | 49.4 |
| PT54978 | 1 | 49.4 |
| PT60155 | 1 | 49.4 |
| PT60613 | 1 | 49.4 |
| C2At3g04780 | 1 | 49.7 |
| C2At2g45910 | 1 | 50 |
| C2At2g38730 | 1 | 50.3 |
| C2At3g18860 | 1 | 51 |
| C2At4g00090 | 1 | 51 |
| PT50645 | 1 | 51 |
| PT50028 | 1 | 52.8 |
| C2At2g16920 | 1 | 54.4 |
| C2At3g08030 | 1 | 56 |
| C2At5g60620 | 1 | 56 |
| C2At4g30890 | 1 | 59.8 |
| PT61218 | 1 | 63 |
| C2At1g18640 | 1 | 68.6 |
| PT61436 | 1 | 71.4 |
| C2At4g03200 | 1 | 74.2 |
| PT51919 | 1 | 77 |
| C2At1g03360 | 1 | 80.8 |
| PT55086 | 1 | 84.6 |
| PT54888 | 1 | 86.4 |
| PT61348 | 1 | 95 |
| PT52848 | 1 | 99.7 |
| PT54845 | 1 | 99.7 |
| PT61286 | 1 | 103.5 |
| C2At5g25760 | 1 | 105.9 |
| C2At5g11810 | 1 | 108.3 |
| C2At1g65700 | 1 | 110.7 |
| C2At3g63200 | 1 | 110.7 |
| C2At2g34560 | 2 | 0 |
| PT61304 | 2 | 0.9 |
| C2At4g20070 | 2 | 1.8 |
| C2At2g35130 | 2 | 2.7 |
| C2At1g30000 | 2 | 3.6 |
| C2At4g18060 | 2 | 4.6 |
| C2At4g21120 | 2 | 4.6 |
| PT52794 | 2 | 4.6 |
| PT54596 | 2 | 4.6 |
| PT61564 | 2 | 4.6 |
| C2At3g02220 | 2 | 5 |
| C2At5g45950 | 2 | 5.4 |
| C2At3g28670 | 2 | 5.8 |
| C2At1g10580 | 2 | 6.2 |
| C2At4g04955 | 2 | 6.2 |
| C2At4g21710 | 2 | 6.2 |
| PT51554 | 2 | 6.2 |
| PT55100 | 2 | 6.2 |
| PT60648 | 2 | 6.2 |
| PT61293 | 2 | 6.2 |
| C2At1g11430 | 2 | 6.7 |
| C2At1g61620 | 2 | 6.7 |
| C2At1g65900 | 2 | 6.7 |
| C2At3g28720 | 2 | 6.7 |
| C2At4g34090 | 2 | 6.7 |
| PT53932 | 2 | 6.7 |
| PT61390 | 2 | 6.7 |
| C2At3g01180 | 2 | 7.3 |
| C2At3g01480 | 2 | 7.3 |
| C2At3g26900 | 2 | 7.3 |
| C2At5g15410 | 2 | 7.3 |
| C2At5g67370 | 2 | 7.3 |
| C2At2g04700 | 2 | 7.9 |
| C2At3g27200 | 2 | 7.9 |
| C2At3g28050 | 2 | 8.5 |
| C2At3g55250 | 2 | 9.8 |
| PT53545 | 2 | 11.1 |
| C2At4g37130 | 2 | 12.4 |
| C2At4g33985 | 2 | 13.7 |
| PT51562 | 2 | 15 |
| PT50692 | 2 | 17.4 |
| PT60665 | 2 | 19.7 |
| PT50693 | 2 | 38.7 |
| C2At1g30580 | 2 | 46.5 |
| PT54378 | 2 | 46.5 |
| C2At5g37260 | 2 | 49.7 |
| C2At1g30540 | 2 | 52.9 |
| C2At3g46780 | 2 | 56.1 |
| C2At1g74730 | 2 | 59.4 |
| PT51247 | 3 | 0 |
| PT52769 | 3 | 0 |
| PT60915 | 3 | 1.6 |
| PT60930 | 3 | 3.2 |
| PT61203 | 3 | 3.2 |
| C2At1g33970 | 3 | 4.7 |
| C2At1g64770 | 3 | 4.7 |
| C2At2g14260 | 3 | 4.7 |
| PT52597 | 3 | 4.7 |
| PT55130 | 3 | 4.7 |
| PT61314 | 3 | 4.7 |
| C2At2g26210 | 3 | 6.3 |
| PT53100 | 3 | 7.9 |
| C2At1g07310 | 3 | 9.4 |
| PT54944 | 3 | 11.3 |
| PT60269 | 3 | 13.1 |
| C2At3g56040 | 3 | 19.7 |
| C2At5g51110 | 3 | 27.4 |
| PT52937 | 3 | 32.1 |
| PT60913 | 3 | 36.8 |
| PT60037 | 3 | 43.4 |
| cLPT2E21 | 3 | 44.4 |
| PT50762 | 3 | 44.4 |
| C2At1g51160 | 3 | 45.4 |
| C2At1g80460 | 3 | 46.4 |
| PT51912 | 3 | 47.4 |
| C2At5g17170 | 3 | 48.4 |
| PT51682 | 3 | 49.4 |
| C2At1g79600 | 3 | 50.4 |
| C2At1g74470 | 3 | 51 |
| C2At3g02910 | 3 | 51 |
| PT50120 | 3 | 51 |
| PT52722 | 3 | 51 |
| PT61454 | 3 | 51 |
| C2At3g03100 | 3 | 51.6 |
| C2At1g67740 | 3 | 52.2 |
| C2At1g80170 | 3 | 52.2 |
| C2At3g48610 | 3 | 52.2 |
| C2At4g39630 | 3 | 52.2 |
| C2At5g13050 | 3 | 52.2 |
| C2At5g63380 | 3 | 52.2 |
| PT51314 | 3 | 52.2 |
| PT53970 | 3 | 52.2 |
| PT60200 | 3 | 52.2 |
| PT60273 | 3 | 52.2 |
| PT61594 | 3 | 52.2 |
| U214603 | 3 | 52.2 |
| C2At1g79720 | 3 | 52.8 |
| PT54245 | 3 | 52.8 |
| C2At3g17970 | 3 | 56 |
| C2At3g18270 | 3 | 60.5 |
| C2At1g80360 | 3 | 70.3 |
| C2At1g52080 | 3 | 82.1 |
| C2At3g13700 | 3 | 89.6 |
| PT52939 | 3 | 89.6 |
| PT50794 | 3 | 96.8 |
| PT54598 | 3 | 96.8 |
| C2At3g14075 | 3 | 100.9 |
| C2At3g14200 | 3 | 106.6 |
| C2At3g21215 | 3 | 111.6 |
| PT60981 | 4 | 0 |
| PT60152 | 4 | 6.3 |
| C2At5g37290 | 4 | 12.5 |
| PT50336 | 4 | 15.3 |
| C2At2g39580 | 4 | 18.1 |
| C2At1g60560 | 4 | 19.9 |
| PT60901 | 4 | 21 |
| C2At3g19895 | 4 | 22 |
| C2At3g51010 | 4 | 27.3 |
| C2At1g68100 | 4 | 31 |
| PT52347 | 4 | 31 |
| PT60172 | 4 | 31 |
| PT60297 | 4 | 37.6 |
| PT54295 | 4 | 40.4 |
| PT60140 | 4 | 41.8 |
| PT50655 | 4 | 43.2 |
| C2At1g44790 | 4 | 43.8 |
| C2At3g17590 | 4 | 43.8 |
| C2At1g05385 | 4 | 44.4 |
| C2At1g21690 | 4 | 45 |
| PT61315 | 4 | 46.9 |
| C2At1g77250 | 4 | 48.8 |
| PT60276 | 4 | 50.7 |
| C2At1g71810 | 4 | 52.7 |
| C2At1g78230 | 4 | 55.8 |
| PT51166 | 4 | 60.9 |
| PT60934 | 4 | 60.9 |
| C2At5g10920 | 4 | 62.9 |
| PT55077 | 4 | 65 |
| PT60053 | 4 | 65 |
| C2At1g46480 | 4 | 70.3 |
| C2At5g25900 | 4 | 75.6 |
| PT50738 | 4 | 77.6 |
| C2At1g19340 | 4 | 78.5 |
| PT53481 | 4 | 79.4 |
| C2At5g42950 | 4 | 83.3 |
| PT60771 | 4 | 87.1 |
| C2At1g20575 | 4 | 89.5 |
| PT50334 | 4 | 91.9 |
| PT61171 | 4 | 91.9 |
| C2At1g42990 | 4 | 94.2 |
| C2At2g46580 | 5 | 0 |
| PT50225 | 5 | 3.8 |
| C2At5g49510 | 5 | 10.4 |
| C2At3g55800 | 5 | 13.7 |
| C2At5g05270 | 5 | 17 |
| C2At5g09380 | 5 | 19.8 |
| C2At3g55360 | 5 | 33.4 |
| PT61226 | 5 | 35.3 |
| PT61396 | 5 | 37.2 |
| C2At3g54860 | 5 | 39 |
| PT53054 | 5 | 43.6 |
| C2At2g01720 | 5 | 45.4 |
| PT61143 | 5 | 45.4 |
| PT60089 | 5 | 47.3 |
| PT61217 | 5 | 47.3 |
| PT61426 | 5 | 47.3 |
| PT50838 | 5 | 49.1 |
| PT53175 | 5 | 49.1 |
| C2At2g28880 | 5 | 51 |
| C2At5g44200 | 5 | 52.8 |
| C2At3g54770 | 5 | 54.7 |
| C2At5g37360 | 5 | 56.5 |
| C2At5g09880 | 5 | 68.1 |
| C2At5g64730 | 5 | 70.9 |
| C2At3g52220 | 5 | 72.7 |
| C2At3g54840 | 5 | 77.3 |
| PT50790 | 5 | 81.1 |
| C2At5g16710 | 5 | 84.9 |
| C2At5g50720 | 5 | 84.9 |
| PT55072 | 5 | 87.9 |
| C2At3g52640 | 5 | 90.9 |
| C2At5g14520 | 5 | 93.9 |
| C2At5g19690 | 6 | 0 |
| PT51085 | 6 | 0 |
| PT51873 | 6 | 0 |
| PT61385 | 6 | 0 |
| PT60606 | 6 | 6 |
| C2At2g32950 | 6 | 12 |
| C2At1g24340 | 6 | 14.7 |
| PT54819 | 6 | 14.7 |
| C2At4g11120 | 6 | 17.2 |
| PT51638 | 6 | 17.2 |
| PT55012 | 6 | 19.7 |
| PT51123 | 6 | 21.6 |
| PT61281 | 6 | 21.6 |
| C2At2g43770 | 6 | 23.4 |
| PT51054 | 6 | 23.4 |
| PT51152 | 6 | 23.4 |
| PT61319 | 6 | 23.4 |
| C2At3g13180 | 6 | 25 |
| C2At4g27700 | 6 | 25 |
| C2At5g62530 | 6 | 26.6 |
| PT53574 | 6 | 28.1 |
| PT53563 | 6 | 31.5 |
| PT52736 | 6 | 34.8 |
| PT60728 | 6 | 34.8 |
| PT53857 | 6 | 36.3 |
| C2At5g22620 | 6 | 37.8 |
| C2At4g34215 | 6 | 43 |
| C2At5g56940 | 6 | 48.2 |
| PT54210 | 6 | 53.4 |
| C2At1g44760 | 6 | 63.5 |
| PT50539 | 6 | 65.3 |
| C2At5g46630 | 6 | 67.2 |
| PT53472 | 6 | 69 |
| PT51132 | 6 | 71.4 |
| C2At4g10030 | 6 | 73.7 |
| PT54322 | 6 | 78.4 |
| C2At1g16870 | 6 | 79.3 |
| C2At1g14850 | 6 | 80.2 |
| PT52760 | 6 | 81.2 |
| PT51913 | 6 | 82.8 |
| PT54484 | 6 | 82.8 |
| PT61467 | 6 | 82.8 |
| C2At1g20050 | 6 | 84.3 |
| PT60603 | 6 | 87.1 |
| C2At4g28530 | 6 | 89.9 |
| PT60129 | 6 | 89.9 |
| C2At2g06010 | 7 | 0 |
| C2At4g26680 | 7 | 0 |
| C2At4g33250 | 7 | 0 |
| PT50311 | 7 | 0 |
| PT50856 | 7 | 0 |
| PT52768 | 7 | 0 |
| PT54079 | 7 | 0 |
| PT60122 | 7 | 0 |
| PT60682 | 7 | 0 |
| PT51241 | 7 | 4.7 |
| C2At2g42750 | 7 | 9.4 |
| PT55194 | 7 | 10.1 |
| C2At3g58790 | 7 | 10.8 |
| PT54184 | 7 | 11.5 |
| C2At2g38020 | 7 | 12.2 |
| C2At3g14910 | 7 | 12.2 |
| C2At1g78600 | 7 | 12.9 |
| C2At1g53670 | 7 | 13.4 |
| C2At1g78620 | 7 | 13.4 |
| C2At1g16900 | 7 | 15.2 |
| PT53203 | 7 | 17.6 |
| C2At3g15290 | 7 | 19.9 |
| C2At3g15380 | 7 | 23.6 |
| C2At5g54310 | 7 | 29.9 |
| C2At1g04970 | 7 | 36.1 |
| C2At1g55840 | 7 | 46.8 |
| C2At5g56130 | 7 | 50.8 |
| C2At4g12740 | 7 | 54.8 |
| C2At5g58410 | 7 | 56.3 |
| PT51706 | 7 | 56.3 |
| PT60870 | 7 | 57.8 |
| C2At2g37500 | 7 | 62.1 |
| C2At3g53580 | 7 | 66.3 |
| PT51779 | 7 | 67.8 |
| PT52378 | 7 | 69.3 |
| C2At2g37025 | 7 | 76.9 |
| PT51575 | 7 | 76.9 |
| PT60291 | 7 | 79.2 |
| PT53216 | 7 | 81.5 |
| C2At3g09920 | 7 | 83.9 |
| C2At3g52610 | 7 | 83.9 |
| C2At3g15410 | 8 | 0 |
| C2At4g03400 | 8 | 0 |
| PT51896 | 8 | 0 |
| PT60677 | 8 | 5.9 |
| PT60212 | 8 | 9.6 |
| PT60165 | 8 | 10.4 |
| TG230 | 8 | 11.2 |
| C2At4g26750 | 8 | 12 |
| C2At3g13235 | 8 | 12.8 |
| PT60849 | 8 | 12.8 |
| PT61214 | 8 | 17.1 |
| C2At2g20860 | 8 | 22 |
| C2At2g46370 | 8 | 26.8 |
| PT52509 | 8 | 26.8 |
| C2At4g19003 | 8 | 28.2 |
| PT50346 | 8 | 29.6 |
| PT51180 | 8 | 29.6 |
| PT52156 | 8 | 32.4 |
| PT60739 | 8 | 32.4 |
| PT60649 | 8 | 33.4 |
| C2At4g32770 | 8 | 34.4 |
| C2At5g11480 | 8 | 35.4 |
| C2At5g11450 | 8 | 36.3 |
| PT53595 | 8 | 36.3 |
| C2At3g53920 | 8 | 45.9 |
| PT61488 | 8 | 55.4 |
| PT51259 | 8 | 59.1 |
| PT52353 | 8 | 60.9 |
| PT52565 | 8 | 62.7 |
| C2At1g20830 | 8 | 64.5 |
| C2At1g32410 | 8 | 66.3 |
| C2At5g47010 | 8 | 66.3 |
| PT53026 | 8 | 68.7 |
| C2At1g62780 | 8 | 71.1 |
| PT53802 | 8 | 73.1 |
| PT53499 | 8 | 75.1 |
| PT53673 | 8 | 75.1 |
| PT60561 | 8 | 75.1 |
| PT61419 | 8 | 75.1 |
| C2At1g12060 | 8 | 77 |
| C2At4g23840 | 8 | 77 |
| C2At5g41350 | 8 | 77 |
| C2At1g16590 | 9 | 0 |
| C2At3g23400 | 9 | 0 |
| C2At5g06360 | 9 | 0 |
| PT51076 | 9 | 13.1 |
| C2At3g24160 | 9 | 15 |
| C2At4g14570 | 9 | 16.8 |
| PT54242 | 9 | 19.6 |
| PT54896 | 9 | 19.6 |
| C2At3g16840 | 9 | 22.8 |
| C2At1g04530 | 9 | 25.9 |
| C2At3g24050 | 9 | 29 |
| C2At3g06790 | 9 | 36 |
| C2At5g18580 | 9 | 42.9 |
| PT53607 | 9 | 43.6 |
| PT60937 | 9 | 43.6 |
| C2At4g15520 | 9 | 44.3 |
| C2At3g23590 | 9 | 45 |
| PT61530 | 9 | 45.6 |
| PT60863 | 9 | 46.2 |
| PT60938 | 9 | 46.8 |
| PT52449 | 9 | 47.4 |
| C2At1g04190 | 9 | 47.9 |
| C2At4g35930 | 9 | 52.9 |
| PT51070 | 9 | 57.9 |
| C2At1g61150 | 9 | 63 |
| C2At1g28530 | 9 | 66.3 |
| cLPT5E7 | 9 | 74.6 |
| E492334 | 9 | 74.6 |
| C2At4g31410 | 10 | 0 |
| PT53522 | 10 | 6 |
| C2At5g04200 | 10 | 11.6 |
| PT51212 | 10 | 27.5 |
| PT52988 | 10 | 30.3 |
| PT60870 | 10 | 31.7 |
| C2At3g51840 | 10 | 33.1 |
| U221455 | 10 | 35.9 |
| PT51339 | 10 | 39.6 |
| PT61494 | 10 | 39.6 |
| C2At3g08760 | 10 | 42.9 |
| C2At5g36210 | 10 | 46.2 |
| PT61457 | 10 | 48.6 |
| C2At1g70160 | 10 | 50.9 |
| C2At4g24750 | 10 | 50.9 |
| PT54871 | 10 | 50.9 |
| PT60675 | 10 | 50.9 |
| PT60877 | 10 | 50.9 |
| C2At1g60440 | 10 | 53.4 |
| C2At1g07040 | 10 | 55.9 |
| C2At1g14270 | 10 | 58.5 |
| C2At2g03510 | 10 | 59.7 |
| C2At1g26520 | 10 | 60.9 |
| C2At5g60990 | 10 | 62.2 |
| PT52640 | 10 | 62.2 |
| PT61583 | 10 | 64.7 |
| C2At1g26670 | 10 | 67.2 |
| PT55265 | 10 | 69.7 |
| C2At1g25580 | 10 | 76.3 |
| C2At2g01110 | 10 | 86.9 |
| PT51967 | 10 | 95.7 |
| C2At2g18710 | 11 | 0 |
| C2At2g24090 | 11 | 8.8 |
| C2At5g58200 | 11 | 16.4 |
| C2At5g06430 | 11 | 36.6 |
| C2At2g06925 | 11 | 37.9 |
| PT54965 | 11 | 39.2 |
| PT61141 | 11 | 40.5 |
| C2At1g56050 | 11 | 42.4 |
| PT51534 | 11 | 42.4 |
| C2At1g26940 | 11 | 52.5 |
| PT50510 | 11 | 62.6 |
| PT60115 | 11 | 66.3 |
| PT50344 | 11 | 69.1 |
| PT53434 | 11 | 69.1 |
| C2At3g44600 | 11 | 71 |
| C2At1g30825 | 11 | 72.9 |
| C2At3g53400 | 11 | 74.8 |
| PT61224 | 11 | 74.8 |
| C2At2g28490 | 11 | 76.8 |
| C2At2g28800 | 11 | 78.7 |
| C2At5g59960 | 11 | 80.6 |
| PT51717 | 11 | 82.5 |
| C2At5g12200 | 11 | 84.5 |
| PT61367 | 11 | 87.3 |
| C2At3g22660 | 11 | 89.1 |
| PT52176 | 11 | 89.1 |
| PT54525 | 11 | 89.1 |
| C2At5g13030 | 11 | 90.6 |
| C2At3g12685 | 11 | 92 |
| PT52935 | 12 | 0 |
| PT60939 | 12 | 12.1 |
| PT61522 | 12 | 16.8 |
| PT51666 | 12 | 20.5 |
| PT60146 | 12 | 20.5 |
| C2At2g24580 | 12 | 21.4 |
| C2At1g14790 | 12 | 22.3 |
| C2At1g30360 | 12 | 23.2 |
| PT61362 | 12 | 24.2 |
| C2At1g22860 | 12 | 26 |
| PT51161 | 12 | 26 |
| C2At1g67325 | 12 | 26.7 |
| C2At1g24360 | 12 | 27.4 |
| C2At1g67700 | 12 | 28.1 |
| C2At1g67730 | 12 | 28.8 |
| PT60228 | 12 | 29.7 |
| PT60572 | 12 | 31.6 |
| C2At5g38530 | 12 | 33.4 |
| C2At4g29735 | 12 | 36.2 |
| PT60205 | 12 | 38.1 |
| C2At4g16580 | 12 | 39.9 |
| PT50226 | 12 | 39.9 |
| C2At1g76150 | 12 | 45.8 |
| PT50989 | 12 | 51.7 |
| C2At1g17410 | 12 | 56.5 |
| C2At3g16290 | 12 | 61.2 |
| PT52906 | 12 | 64.2 |
| PT54772 | 12 | 67.9 |
| C2At3g17000 | 12 | 69.5 |
| C2At5g21170 | 12 | 71.1 |
| PT52058 | 12 | 71.1 |
| PT53009 | 12 | 71.1 |
| C2At1g48300 | 12 | 72.8 |
| C2At5g53000 | 12 | 79.9 |
| PT54576 | 12 | 86.9 |
