# Supplementary material for: Reference genomes and transcriptomes of Nicotiana sylvestris and Nicotiana tomentosiformis
Source: Genome Biol. 2013 Jun 17;14(6):R60. doi: 10.1186/gb-2013-14-6-r60 (PMC3707018; doi:10.1186/gb-2013-14-6-r60)
Supplement: Additional file 8 — Comparisons between the COSII mapping to the draft genomes and existing genetic maps. [file gb-2013-14-6-r60-S8.DOCX]

Additional file 8: Comparisons between the COSII mapping to the draft genomes and existing genetic maps.

|  | Assemblies | | | |
| --- | --- | --- | --- | --- |
| Genetic map | *N. sylvestris* | *N. tomentosiformis* | *N. sylvestris* and  *N. tomentosiformis* | None |
| *N. acuminata* | 1 (4%) | 2 (8%) | 5 (19%) | 18 (69%) |
| *N. tomentosiformis* | 13 (8%) | 16 (10%) | 31 (20%) | 94 (61%) |
| *N. acuminata* and *N. tomentosiformis* | 10 (9%) | 5 (5%) | 29 (27%) | 64 (59%) |
| Unknown | 68 (6%) | 78 (6%) | 226 (18%) | 861 (70%) |
