# Supplementary material for: Reference genomes and transcriptomes of Nicotiana sylvestris and Nicotiana tomentosiformis
Source: Genome Biol. 2013 Jun 17;14(6):R60. doi: 10.1186/gb-2013-14-6-r60 (PMC3707018; doi:10.1186/gb-2013-14-6-r60)
Supplement: Additional file 9 — Statistics of the Nicotiana sylvestris and Nicotiana tomentosiformis RNA-seq libraries. [file gb-2013-14-6-r60-S9.DOCX]

Additional file 9:

Table 9: Number of read pairs obtained for each sample. NB: for technical reasons a further sample from a different biological replicate was used to generate the *N. tomentosiformis* flower transcripts, than was used for the quantitative assessment (“replicate X”).

| **Tissue** | **Replicate** | ***N. sylvestris*** | ***N. tomentosiformis*** |
| --- | --- | --- | --- |
| Flower | 1 | 108,436,707 | 81,952,371 |
|  | 2 | 119,963,865 | 136,125,112 |
|  | 3 | 67,726,775 | 159,926,922 |
|  | X |  | 61,176,439 |
| Leaf | 1 | 56,623,372 | 68,790,307 |
|  | 2 | 81,624,574 | 49,314,799 |
|  | 3 | 60,897,419 | 79,977,203 |
| Root | 1 | 86,993,889 | 80,974,445 |
|  | 2 | 82,462,722 | 89,277,997 |
|  | 3 | 83,811,950 | 100,085,711 |
