# Supplementary material for: Reference genomes and transcriptomes of Nicotiana sylvestris and Nicotiana tomentosiformis
Source: Genome Biol. 2013 Jun 17;14(6):R60. doi: 10.1186/gb-2013-14-6-r60 (PMC3707018; doi:10.1186/gb-2013-14-6-r60)
Supplement: Additional file 10 — Transcriptome sequence number and length distribution. [file gb-2013-14-6-r60-S10.DOCX]

Additional file 10. Transcriptome sequence number and length distribution (quartiles Q0 through Q4).

|  | | ***N. sylvestris*** | | | ***N. tomentosiformis*** | | |
| --- | --- | --- | --- | --- | --- | --- | --- |
|  |  | Mapping (merging) | de novo | Mapping (merging) | | de-novo |  |
| Transcripts (RNA) | Number of sequences | 68779 | 249849 | 66046 | | 262822 |  |
|  | Q0 | 72.00 | 100 | 76.00 | | 100 |  |
|  | Q1 | 886.00 | 143 | 910.00 | | 145 |  |
|  | Q2 | 1530.00 | 255 | 1570.00 | | 259 |  |
|  | Q3 | 2476.50 | 688 | 2503.75 | | 663 |  |
|  | Q4 | 24875.00 | 16722 | 28169.00 | | 20533 |  |
| Predicted proteins | Number of sequences | 38940 | 39267 | 38648 | | 42811 |  |
|  | Q0 | 100 | 100 | 100 | | 100 |  |
|  | Q1 | 198 | 155 | 198 | | 148 |  |
|  | Q2 | 323 | 258 | 321 | | 241 |  |
|  | Q3 | 501 | 431 | 500 | | 410 |  |
|  | Q4 | 5101 | 5428 | 5102 | | 5430 |  |
