# Supplementary material for: Reference genomes and transcriptomes of Nicotiana sylvestris and Nicotiana tomentosiformis
Source: Genome Biol. 2013 Jun 17;14(6):R60. doi: 10.1186/gb-2013-14-6-r60 (PMC3707018; doi:10.1186/gb-2013-14-6-r60)
Supplement: Additional file 11 — Genomic GO term (Biological Process) enrichment in Nicotiana sylvestris and Nicotiana tomentosiformis genes. [file gb-2013-14-6-r60-S11.DOCX]

Additional file 11

**Table 1: Genomic GO term (Biological Process) enrichment in Nicotiana sylvestris genes.**

| **#** | **GOBPID** | **Pvalue** | **ExpCount** | **Count** | **Term** |
| --- | --- | --- | --- | --- | --- |
| 1 | GO:0006952 | 0.023874 | 368.1911 | 395 | defense response |
| 2 | GO:0042430 | 0.046947 | 20.23028 | 26 | indole-containing compound metabolic process |
| 3 | GO:0006568 | 0.046947 | 20.23028 | 26 | tryptophan metabolic process |
| 4 | GO:0006586 | 0.046947 | 20.23028 | 26 | indolalkylamine metabolic process |
| 5 | GO:0015991 | 0.048685 | 41.97783 | 50 | ATP hydrolysis coupled proton transport |
| 6 | GO:0015988 | 0.048685 | 41.97783 | 50 | energy coupled proton transport, against electrochemical gradient |

**Table 2: GO term enrichment (Biological process) in Nicotiana tomentosiformis genes.**

| **#** | **GOBPID** | **Pvalue** | **ExpCount** | **Count** | **Term** |
| --- | --- | --- | --- | --- | --- |
| 1 | GO:0006793 | 0.012623 | 1777.298 | 1840 | phosphorus metabolic process |
| 2 | GO:0006796 | 0.012623 | 1777.298 | 1840 | phosphate metabolic process |
| 3 | GO:0044237 | 0.021339 | 5963.536 | 6045 | cellular metabolic process |
| 4 | GO:0016310 | 0.022018 | 1636.439 | 1691 | phosphorylation |
| 5 | GO:0015914 | 0.023702 | 27.18337 | 35 | phospholipid transport |
| 6 | GO:0006468 | 0.023757 | 1578.118 | 1631 | protein phosphorylation |
| 7 | GO:0044260 | 0.027042 | 4354.281 | 4428 | cellular macromolecule metabolic process |
| 8 | GO:0043412 | 0.030073 | 2089.659 | 2146 | macromolecule modification |
| 9 | GO:0018202 | 0.03057 | 35.5855 | 44 | peptidyl-histidine modification |
| 10 | GO:0043170 | 0.039886 | 4978.016 | 5047 | macromolecule metabolic process |
| 11 | GO:0018106 | 0.042461 | 31.63155 | 39 | peptidyl-histidine phosphorylation |
