# Supplementary material for: Reference genomes and transcriptomes of Nicotiana sylvestris and Nicotiana tomentosiformis
Source: Genome Biol. 2013 Jun 17;14(6):R60. doi: 10.1186/gb-2013-14-6-r60 (PMC3707018; doi:10.1186/gb-2013-14-6-r60)
Supplement: Additional file 12 — Non-exhaustive list of gene copies potentially involved in cadmium/zinc (Cd/Zn) accumulation in Nicotiana leaves. [file gb-2013-14-6-r60-S12.DOCX]

Additional file 12. Non-exhaustive list of gene copies potentially involved in cadmium/zinc (Cd/Zn) accumulation in Nicotiana leaves.

Syl: *N. sylvestris*; Tom: *N. tomentosiformis*; IRT: iron transporter protein; HMA: heavy metal ATPase; PCS: phytochelatin synthase; NS: nicotinamine synthase; PDR: pleiotropic drug resistance; MRP: multidrug resistance-associated protein; ATM: ABC transporter of the mitochondrion; CAX: cation/proton exchanger. The numbers of gene specific ExonArray probes and the numbers of probes (in parenthesis) that are also species-specific are given. Specific Affymetrix probes (Tobacco exon array, with 100% matches to annotated sequences) were challenged with RNA isolated from leaf of *N. sylvestris* and *N. tomentosiformis* plantlets grown on floating tray for three weeks. The array data (log 2 expression) are reported in the right columns: 0: log 2values below 3; *: log2 values between 3–5; **: log2 values between 5–7; ***: log2 values between 7–9; n.d.: not detected.

| Gene family | Number | FPKM expression | | | | | | | | | ExonArray probes | ExonArray expression in leaf |
| --- | --- | --- | --- | --- | --- | --- | --- | --- | --- | --- | --- | --- |
|  |  | Root | | | Leaf | | | Flower | | |  |  |
|  |  | R1 | R2 | R3 | L1 | L2 | L3 | F1 | F2 | F3 |  |  |
| ***Nicotiana tomentosiformis*** | | | | | | | | | | | | |
| IRT | 3 | 0 ± 0 | 0 ± 0 | 0 ± 0 | 0 ± 0 | 0 ± 0 | 0 ± 0 | 0 ± 0 | 0 ± 0 | 0 ± 0 | 2 (2) | 0 |
|  |  | 0 ± 0 | 0 ± 0 | 0 ± 0 | 0 ± 0 | 0 ± 0 | 0 ± 0 | 0 ± 0 | 0 ± 0 | 0 ± 0 | 3 (3) | 0 |
|  |  | 0 ± 0 | 0 ± 0 | 0 ± 0 | 0 ± 0 | 0 ± 0 | 0 ± 0 | 6.3 ± 0.6 | 5.8 ± 0.4 | 7.5 ± 0.7 | 3 (2) | 0 |
| HMA | 1 | 48.5 ± 0.7 | 55.4 ± 0.8 | 40.7 ± 0.6 | 11.3 ± 0.4 | 12.9 ± 0.5 | 9.9 ± 0.4 | 35.2 ± 0.6 | 34.3 ± 0.5 | 30.1 ± 0.4 | 4 (2) | * |
| PCS | 1 | 46.9 ± 1.1 | 49.1 ± 1.3 | 47.4 ± 1 | 31.1 ± 1 | 22.4 ± 0.9 | 27.9 ± 0.8 | 21.1 ± 0.7 | 21 ± 0.5 | 18.9 ± 0.5 | 5 (3) | ** |
| NS | 2 | 6.3 ± 0.5 | 9.6 ± 0.6 | 3.1 ± 0.3 | 39.3 ± 2.6 | 84.9 ± 2.3 | 21.6 ± 0.9 | 7.7 ± 0.6 | 6.1 ± 0.4 | 6.4 ± 0.3 | 1 (1) | ** |
|  |  | 0.5 ± 0.2 | 0.5 ± 0.2 | 0.2 ± 0.1 | 0 ± 0 | 0 ± 0 | 0 ± 0 | 0 ± 0 | 0 ± 0 | 0.2 ± 0.1 | 1(1) | 0 |
| PDR | 1 | 13 ± 0.4 | 11.2 ± 0.3 | 9.8 ± 0.3 | 16.2 ± 0.4 | 12 ± 0.4 | 26.8 ± 1.3 | 11.5 ± 0.3 | 22.7 ± 0.4 | 14 ± 0.3 | 7 (3) | * |
| MRP | 1 | 81.2 ± 0.9 | 70.2 ± 0.8 | 70.9 ± 0.7 | 1.1 ± 0.1 | 1.4 ± 0.1 | 0.8 ± 0.1 | 8.8 ± 0.3 | 14.2 ± 0.3 | 9.4 ± 0.2 | 4 (3) | *** |
| ATM | 1 | 19.4 ± 0.6 | 25.9 ± 1.1 | 24.8 ± 0.8 | 18.4 ± 0.8 | 16.6 ± 0.8 | 30.7 ± 2.9 | 11.6 ± 0.5 | 12.3 ± 0.4 | 13 ± 0.4 | 3 (1) | *** |
| CAX | 4 | 17.1 ± 0.8 | 20.3 ± 0.8 | 20.6 ± 0.7 | 18.9 ± 0.9 | 37.5 ± 2.9 | 16.1 ± 0.7 | 19.4 ± 0.8 | 17 ± 0.5 | 20.9 ± 0.6 | 10 (6) | * |
|  |  | 10.9 ± 0.6 | 12.7 ± 0.6 | 8.7 ± 0.5 | 11.7 ± 0.7 | 11.6 ± 1.8 | 12.5 ± 1.1 | 8.1 ± 0.5 | 6.9 ± 0.4 | 10.8 ± 0.9 | 1 (0) | n.d. |
|  |  | 4.7 ± 0.4 | 4.7 ± 0.3 | 5.4 ± 0.3 | 3.9 ± 0.4 | 4.5 ± 0.4 | 4.8 ± 0.6 | 6.3 ± 0.4 | 13.4 ± 0.7 | 7.6 ± 0.3 | 2 (2) | * |
|  |  | 0 ± 0 | 0 ± 0 | 0 ± 0 | 0 ± 0 | 0 ± 0 | 0 ± 0 | 0 ± 0 | 0 ± 0 | 0 ± 0 | 5 (4) | 0 |
| ***Nicotiana sylvestris*** | | | | | | | | | | | | |
| IRT | 3 | 11.1 ± 0.7 | 32.3 ± 1.3 | 40.8 ± 1.4 | 0 ± 0 | 0 ± 0 | 0 ± 0 | 0 ± 0 | 0 ± 0 | 0 ± 0 | 3 (3) | 0 |
|  |  | 1.4 ± 0.3 | 3.4 ± 0.4 | 4.5 ± 0.5 | 0 ± 0 | 0 ± 0 | 0 ± 0 | 0 ± 0 | 0 ± 0 | 0 ± 0 | 2 (2) | 0 |
|  |  | 0 ± 0 | 0 ± 0 | 0 ± 0 | 0 ± 0 | 0 ± 0 | 0 ± 0 | 48.7 ± 1.2 | 21.8 ± 0.6 | 66.7 ± 1.7 | 3 (2) | 0 |
| HMA | 1 | 83.7 ± 1.1 | 77 ± 1.2 | 86.7 ± 1.2 | 11.4 ± 0.4 | 11.8 ± 0.4 | 13.6 ± 0.7 | 13.2 ± 0.3 | 18.9 ± 0.4 | 14.5 ± 0.4 | 15 (13) | * |
| PCS | 1 | 14 ± 0.7 | 14.6 ± 0.6 | 13.1 ± 0.5 | 15.3 ± 0.9 | 15 ± 0.7 | 16.5 ± 0.7 | 12.6 ± 0.5 | 12.5 ± 0.5 | 13.8 ± 0.7 | 2 (0) | n.d. |
| NS | 2 | 23.4 ± 0.9 | 30.6 ± 1.1 | 42.1 ± 1.3 | 35.1 ± 1.4 | 42.4 ± 1.3 | 45.2 ± 1.5 | 9.8 ± 0.5 | 17.9 ± 0.7 | 4.8 ± 0.5 | 0 (0) | n.d. |
|  |  | 9.7 ± 0.6 | 14.5 ± 0.8 | 19.6 ± 0.9 | 0 ± 0 | 0 ± 0 | 0 ± 0 | 1.7 ± 0.2 | 1.4 ± 0.2 | 0.9 ± 0.2 | 1 (1) | 0 |
| PDR | 1 | 113.1 ± 1 | 101.1 ± 1 | 129 ± 1.1 | 1.1 ± 0.2 | 0.9 ± 0.1 | 1.3 ± 0.5 | 13.5 ± 0.4 | 8.8 ± 0.2 | 9.8 ± 0.3 | 6 (2) | * |
| MRP | 1 | 40.9 ± 1.4 | 15 ± 0.4 | 15.8 ± 0.4 | 16.5 ± 0.6 | 15.3 ± 0.4 | 33.9 ± 1.6 | 4.9 ± 0.2 | 6.4 ± 0.5 | 5.8 ± 0.3 | 3 (2) | ** |
| ATM | 1 | 10.8 ± 0.5 | 11.5 ± 0.6 | 12.1 ± 0.5 | 9.7 ± 0.7 | 10 ± 0.5 | 8.6 ± 0.6 | 10.2 ± 0.4 | 10.7 ± 0.4 | 10.6 ± 0.5 | 8 (6) | *** |
| CAX | 4 | 24.7 ± 0.9 | 28.1 ± 0.9 | 25.6 ± 0.9 | 17.3 ± 0.9 | 17.4 ± 0.7 | 30 ± 6 | 17.7 ± 0.6 | 17.3 ± 0.6 | 18.6 ± 0.8 | 7 (3) | ** |
|  |  | 33.3 ± 1 | 38.8 ± 1.2 | 34.3 ± 1 | 21.6 ± 1.3 | 17.1 ± 0.9 | 31 ± 1.5 | 16.5 ± 0.6 | 16.6 ± 0.6 | 16.8 ± 1 | 4 (3) | ** |
|  |  | 2.7 ± 0.3 | 1.3 ± 0.2 | 1.3 ± 0.2 | 2 ± 0.3 | 2.7 ± 0.3 | 4.7 ± 0.9 | 7.6 ± 0.4 | 5 ± 0.3 | 7.1 ± 0.5 | 2 (2) | * |
|  |  | 0.5 ± 0.2 | 1.2 ± 0.7 | 0.5 ± 0.2 | 0 ± 0 | 0 ± 0 | 0.2 ± 0.1 | 1.5 ± 0.3 | 1 ± 0.2 | 2.3 ± 0.5 | 3 (2) | 0 |
