# Supplementary material for: Reference genomes and transcriptomes of Nicotiana sylvestris and Nicotiana tomentosiformis
Source: Genome Biol. 2013 Jun 17;14(6):R60. doi: 10.1186/gb-2013-14-6-r60 (PMC3707018; doi:10.1186/gb-2013-14-6-r60)
Supplement: Additional file 13 — Phylogenetic tree of CAX proteins from the N. sylvestris, N. tomentosiformis and Arabidopsis genomes. The N. sylvestris and N. tomentosiformis proteins are numbered according to the rows of Additional file 12. Bootstrap percentages are shown at each node. [file gb-2013-14-6-r60-S13.DOCX]

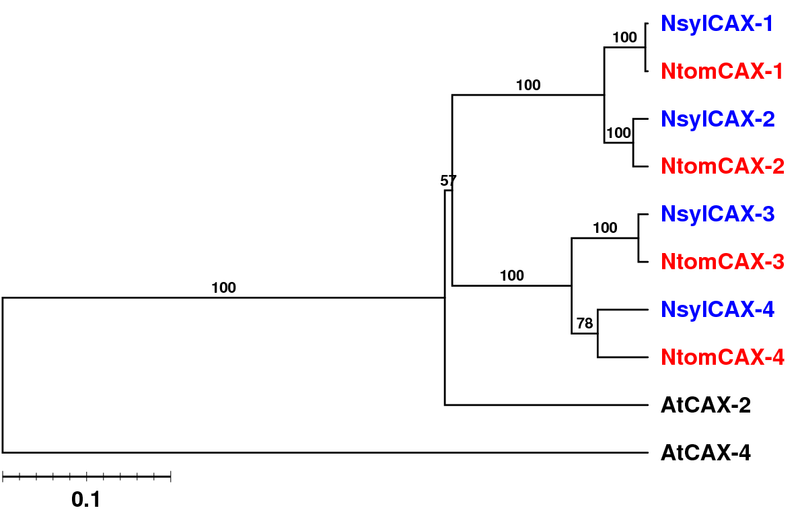


Additional file 13: Phylogenetic tree of CAX proteins from the *N. sylvestris*, *N. tomentosiformis* and Arabidopsis genomes. AtCAX-2: TAIR10 accession AT3G13320.1; AtCAX-4: TAIR10 accession AT5G01490.1. The *N. sylvestris* and *N. tomentosiformis* proteins are numbered according to the rows in Additional file 12. Bootstrap percentages are shown at each node.
