# Supplementary material for: Reference genomes and transcriptomes of Nicotiana sylvestris and Nicotiana tomentosiformis
Source: Genome Biol. 2013 Jun 17;14(6):R60. doi: 10.1186/gb-2013-14-6-r60 (PMC3707018; doi:10.1186/gb-2013-14-6-r60)
Supplement: Additional file 14 — Key genes involved in the synthesis of nicotine and nornicotine alkaloids in Nicotiana leaves. [file gb-2013-14-6-r60-S14.DOCX]

Additional file 14. Key genes involved in the synthesis of nicotine and nornicotine alkaloids in Nicotiana leaves. Syl: *N. sylvestris*; Tom: *N. tomentosiformis*; R: root; L: leaf; F: flower; AO: L-aspartate oxidase; QS: quinolinate synthase; QPT: quinolinate phosphoribosyltransferase; BBL: berberine bridge enzyme-like protein; PMT: putrecine N-methyltransferase; MPO: methyleputrescine oxidase; NND: nicotine N-demethylase. The numbers of gene specific ExonArray probes and the numbers of probes (in parenthesis) that are also species-specific are given. Specific Affymetrix probes (Tobacco exon array, 100% matching with annotated sequences) were challenged with RNA isolated from leaf of *N. sylvestris* and *N. tomentosiformis* plantlets grown on floating tray for three weeks. The array data (log 2 expression) are reported on the right columns: 0: log 2values below 3; *: log2 values between 3–5; **: log2 values between 5–7; ***: log2 values between 7–9; n.d.: not detected.

| Gene family | Number | FPKM expression | | | | | | | | | ExonArray probes | ExonArray expression in leaf |
| --- | --- | --- | --- | --- | --- | --- | --- | --- | --- | --- | --- | --- |
|  |  | Root | | | Leaf | | | Flower | | |  |  |
|  |  | R1 | R2 | R3 | L1 | L2 | L3 | F1 | F2 | F3 |  |  |
| ***Nicotiana tomentosiformis*** | | | | | | | | | | | | |
| AO | 2 | 11.2 ± 0.3 | 14.8 ± 0.4 | 13.2 ± 0.3 | 8.3 ± 0.6 | 8 ± 0.8 | 10.9 ± 0.7 | 14.7 ± 0.8 | 14.2 ± 0.5 | 4.4 ± 0.2 | 1 (1) | * |
|  |  | 4.5 ± 0.3 | 1.6 ± 0.2 | 2.5 ± 0.2 | 0.7 ± 0.3 | 0.6 ± 0.3 | 0.7 ± 0.3 | 0.3 ± 0.1 | 0.3 ± 0.1 | 0.2 ± 0.1 | 9 (7) | * |
| QS | 1 | 59.9 ± 1.1 | 37.7 ± 0.8 | 34.5 ± 0.7 | 11.1 ± 0.5 | 9.7 ± 0.5 | 11.5 ± 0.5 | 19.4 ± 0.6 | 25 ± 0.5 | 14.9 ± 0.4 | 4 (3) | ** |
| QPT | 2 | 34.2 ± 1.1 | 26 ± 0.9 | 21.7 ± 0.8 | 38.2 ± 1.3 | 33.7 ± 1.8 | 37.3 ± 1.2 | 71.2 ± 1.6 | 86.5 ± 1.3 | 65.2 ± 1.6 | 3 (1) | 0 |
|  |  | 17.7 ± 1 | 22.3 ± 1.4 | 17.6 ± 0.8 | 23.7 ± 1.2 | 20.6 ± 1.2 | 22.1 ± 0.9 | 17.5 ± 0.9 | 16.8 ± 0.7 | 14.1 ± 0.6 | 8 (7) | ** |
| A622 | 1 | 57.1 ± 1.6 | 37.3 ± 1.2 | 33.6 ± 1.1 | 0 ± 0 | 0 ± 0 | 0 ± 0 | 0 ± 0 | 0.3 ± 0.1 | 0.2 ± 0.1 | 4 (1) | 0 |
| BBL | 3 | 73 ± 1.4 | 50.1 ± 1.1 | 70.4 ± 1.2 | 0 ± 0 | 0 ± 0 | 0 ± 0 | 3 ± 0.3 | 1.1 ± 0.1 | 2 ± 0.2 | 1 (1) | 0 |
|  |  | 10.5 ± 1.1 | 0.2 ± 0.1 | 0 ± 0 | 0.5 ± 0.1 | 0.5 ± 0.1 | 0.6 ± 0.1 | 0 ± 0 | 0.1 ± 0.1 | 0.1 ± 0 | 4 (4) | * |
|  |  | 11.7 ± 0.9 | 7 ± 0.4 | 7.8 ± 0.4 | 5.6 ± 0.3 | 6 ± 0.3 | 5.3 ± 0.2 | 2.3 ± 0.2 | 1.6 ± 0.1 | 1.6 ± 0.1 | 1 (1) | 0 |
| PMT | 2 | 0 ± 0 | 0 ± 0 | 0 ± 0 | 0 ± 0 | 0 ± 0 | 0 ± 0 | 0 ± 0 | 0.3 ± 0.1 | 0 ± 0 | 0 (0) | n.d. |
|  |  | 0 ± 0 | 0.8 ± 0.3 | 0 ± 0 | 0 ± 0 | 0 ± 0 | 0 ± 0 | 0 ± 0 | 0 ± 0 | 0 ± 0 | 0 (0) | n.d. |
| MPO | 2 | 65.8 ± 1.1 | 55.1 ± 0.9 | 71 ± 1.2 | 0 ± 0 | 0 ± 0 | 0 ± 0 | 1.6 ± 0.2 | 0.2 ± 0.1 | 0.6 ± 0.1 | 8 (2) | 0 |
|  |  | 3.8 ± 0.3 | 3.4 ± 0.3 | 4.9 ± 0.3 | 0.6 ± 0.1 | 0.6 ± 0.1 | 0.6 ± 0.1 | 2.4 ± 0.2 | 2.3 ± 0.2 | 2 ± 0.1 | 9 (7) | * |
| NND | 4 | 109 ± 4.3 | 90.5 ± 2.3 | 117.5 ± 4.3 | 92.4 ± 3.8 | 63.7 ± 3.4 | 70.3 ± 2.8 | 1722.3 ± 7.5 | 1391.2 ± 5.4 | 2600.6 ± 6.8 | 2 (2) | 0 |
|  |  | 63.9 ± 2.7 | 17.1 ± 0.7 | 61.7 ± 2.6 | 32.2 ± 1 | 111.8 ± 9.5 | 43.6 ± 1 | 161 ± 1.9 | 59.6 ± 0.6 | 204.3 ± 1.7 | 1 (1) | 0 |
|  |  | 227.2 ± 3 | 267.7 ± 3.1 | 192.8 ± 2.5 | 45.3 ± 1.4 | 67.1 ± 2 | 46.3 ± 1.3 | 61.8 ± 1.6 | 32.1 ± 0.9 | 52.8 ± 1 | 1 (1) | ** |
|  |  | 0 ± 0 | 0 ± 0 | 0 ± 0 | 0 ± 0 | 0 ± 0 | 0 ± 0 | 2.7 ± 0.3 | 11.5 ± 0.5 | 0 ± 0 | 0 (0) | n.d. |
| ***Nicotiana sylvestris*** | | | | | | | | | | | | |
| AO | 2 | 15.2 ± 1.2 | 18.1 ± 2.7 | 13.1 ± 1.4 | 31.9 ± 3.1 | 15.7 ± 0.4 | 39.4 ± 2.1 | 14 ± 1.4 | 13.5 ± 1.3 | 9.9 ± 0.6 | 6 (4) | * |
|  |  | 193.9 ± 2 | 244 ± 5.1 | 222.5 ± 4.2 | 0.2 ± 0.1 | 0.5 ± 0.2 | 0 ± 0 | 1.3 ± 0.2 | 1.7 ± 0.2 | 1.4 ± 0.2 | 2 (2) | 0 |
| QS | 1 | 333.8 ± 2.4 | 341.5 ± 2.5 | 318.6 ± 2.4 | 24.1 ± 0.8 | 24.1 ± 0.7 | 25.3 ± 0.8 | 25.6 ± 0.6 | 32.3 ± 0.6 | 25.5 ± 0.7 | 2 (1) | *** |
| QPT | 2 | 326 ± 3.6 | 324.5 ± 4 | 265.8 ± 3.2 | 24.3 ± 1.2 | 26.7 ± 1.3 | 32 ± 2.1 | 39.3 ± 1.4 | 34.6 ± 0.9 | 33.9 ± 1.2 | 4 (1) | ** |
|  |  | 20.5 ± 0.9 | 23.6 ± 1.4 | 19.1 ± 0.8 | 26.4 ± 1.3 | 24.4 ± 1 | 21.1 ± 1 | 27.1 ± 0.9 | 28 ± 1.1 | 25.6 ± 1.1 | 1 (0) | n.d. |
| A622 | 1 | 339.8 ± 3.7 | 444.9 ± 4.2 | 331.5 ± 3.6 | 0 ± 0 | 0 ± 0 | 0 ± 0 | 0.7 ± 0.1 | 0.9 ± 0.2 | 0.7 ± 0.2 | 10 (8) | 0 |
| BBL | 4 | 124.1 ± 1.8 | 159.1 ± 2 | 128.4 ± 1.9 | 0.3 ± 0.1 | 0.3 ± 0.1 | 0.3 ± 0.2 | 8.5 ± 0.4 | 11.1 ± 0.5 | 8.2 ± 0.5 | 1 (1) | 0 |
|  |  | 0 ± 0 | 0.2 ± 0.1 | 0.2 ± 0.1 | 1 ± 0.5 | 1 ± 0.1 | 0.4 ± 0.1 | 3 ± 0.2 | 0 ± 0 | 0.4 ± 0.1 | 3 (3) | * |
|  |  | 4.1 ± 0.3 | 2.9 ± 0.3 | 4.1 ± 0.3 | 0 ± 0 | 0 ± 0 | 0 ± 0 | 0.5 ± 0.1 | 0.4 ± 0.1 | 0.5 ± 0.1 | 1 (1) | 0 |
|  |  | 2.4 ± 0.3 | 2.5 ± 0.6 | 2.1 ± 0.3 | 0 ± 0 | 0 ± 0 | 0 ± 0 | 0.3 ± 0.1 | 0.2 ± 0.1 | 0.3 ± 0.1 | 2 (2) | 0 |
| PMT | 3 | 226.4 ± 3.9 | 213.2 ± 3 | 178.5 ± 2.7 | 0.3 ± 0.2 | 0 ± 0 | 0 ± 0 | 0 ± 0 | 0 ± 0 | 0 ± 0 | 2 (2) | 0 |
|  |  | 226.4 ± 3.9 | 71.1 ± 1.9 | 0 ± 0 | 0 ± 0 | 0 ± 0 | 0 ± 0 | 0 ± 0 | 0 ± 0 | 0 ± 0 | 3 (3) | 0 |
|  |  | 20.6 ± 0.9 | 28.1 ± 1.5 | 17.5 ± 0.8 | 0 ± 0 | 0 ± 0 | 0 ± 0 | 0.9 ± 0.2 | 0.5 ± 0.1 | 0.7 ± 0.2 | 3 (3) | 0 |
| MPO | 2 | 54.8 ± 1 | 57.3 ± 1.2 | 57.9 ± 1 | 0 ± 0 | 0 ± 0 | 0 ± 0 | 0.8 ± 0.1 | 3.7 ± 0.2 | 0.2 ± 0.1 | 13 (7) | 0 |
|  |  | 1 ± 0.1 | 1.6 ± 0.2 | 1.3 ± 0.2 | 0 ± 0 | 0 ± 0 | 0 ± 0 | 2 ± 0.2 | 2 ± 0.2 | 1.8 ± 0.2 | 3 (1) | 0 |
| NND | 5 | 251.7 ± 3 | 199.8 ± 2.6 | 184.6 ± 2.6 | 21.6 ± 1.1 | 18.5 ± 0.8 | 25.5 ± 1.1 | 26.3 ± 0.8 | 39.3 ± 1.1 | 25.3 ± 1 | 2 (2) | * |
|  |  | 0 ± 0 | 0 ± 0 | 0 ± 0 | 52 ± 2.1 | 68.8 ± 2 | 87.9 ± 2.5 | 11.9 ± 0.9 | 14.3 ± 0.9 | 16.3 ± 1.3 | 3 (3) | * |
|  |  | 0.7 ± 0.2 | 0.6 ± 0.3 | 0.4 ± 0.2 | 0 ± 0 | 0 ± 0 | 83.9 ± 36.6 | 22.3 ± 1 | 121.2 ± 2.2 | 18.3 ± 1.1 | 0 (0) | n.d. |
|  |  | 0 ± 0 | 0 ± 0 | 0.4 ± 0.2 | 0 ± 0 | 0 ± 0 | 83.9 ± 36.6 | 0.1 ± 0.1 | 0.4 ± 0.2 | 0.5 ± 0.2 | 1 (1) | n.d. |
|  |  | 0 ± 0 | 0 ± 0 | 0 ± 0 | 4.3 ± 0.6 | 3.7 ± 0.5 | 4.8 ± 0.6 | 0 ± 0 | 0 ± 0 | 0 ± 0 | 0 (0) | n.d. |
