# Supplementary material for: Reference genomes and transcriptomes of Nicotiana sylvestris and Nicotiana tomentosiformis
Source: Genome Biol. 2013 Jun 17;14(6):R60. doi: 10.1186/gb-2013-14-6-r60 (PMC3707018; doi:10.1186/gb-2013-14-6-r60)
Supplement: Additional file 15 — Phylogenetic tree of PMT proteins from the N. sylvestris, N. tomentosiformis and N. tabacum genomes. The N. sylvestris and N. tomentosiformis proteins are numbered according to the rows of Additional file 14. Bootstrap percentages are shown at each node. [file gb-2013-14-6-r60-S15.DOCX]

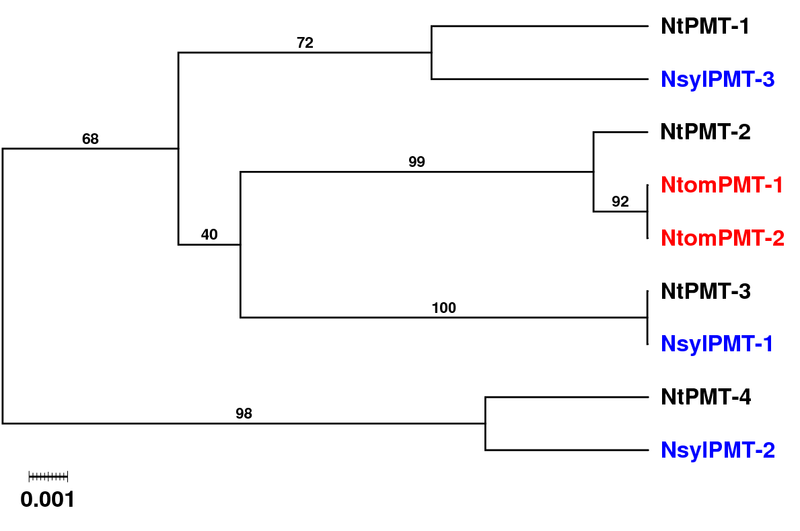


Additional file 15: Phylogenetic tree of PMT proteins from the *N. sylvestris*, *N. tomentosiformis* and *N. tabacum* genomes. NtPMT-1: Genbank accession AAF14881.1; NtPMT-2: Genbank accession AAF14879.1; NtPMT-3: Genbank accession AAF14880.1; NtPMT-4: Genbank accession AAF14878.1. The *N. sylvestris* and *N. tomentosiformis* proteins are numbered according to the rows of Additional file 14. Bootstrap percentages are shown at each node.
