# Supplementary material for: Reference genomes and transcriptomes of Nicotiana sylvestris and Nicotiana tomentosiformis
Source: Genome Biol. 2013 Jun 17;14(6):R60. doi: 10.1186/gb-2013-14-6-r60 (PMC3707018; doi:10.1186/gb-2013-14-6-r60)
Supplement: Additional file 16 — Phylogenetic tree of CYP82E cytochrome P450 proteins and orthologs from the N. sylvestris and N. tomentosiformis genomes. The N. sylvestris and N. tomentosiformis proteins are numbered according to the rows of Additional file 14. Bootstrap percentages are shown at each node. [file gb-2013-14-6-r60-S16.DOCX]

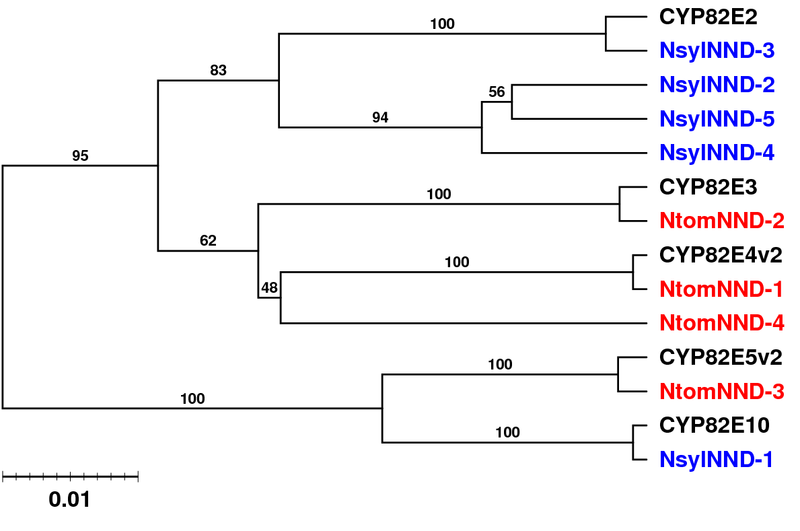


Additional file 16: Phylogenetic tree of CYP82E cytochrome P450 proteins and orthologs from the *N. sylvestris* and *N. tomentosiformis* genomes. CYP82E2: UniProt accession Q38Q85; CYP82E3: UniProt accession Q38Q84; CYP82E4v2: UniProt accession Q38Q87; CYP82E5v2: UniProt accession A9QNE6; CYP82E10: UniProt accession E5G962; The *N. sylvestris* and *N. tomentosiformis* proteins are numbered according to the rows of Additional file 14. Bootstrap percentages are shown at each node.
